# Effect of operational parameters on performance of a self-propelled battery-operated leafy vegetable harvester for fenugreek (*Trigonella foenum-graecum* L.)

**DOI:** 10.1038/s41598-026-50874-8

**Published:** 2026-08-20

**Authors:** Kalluri Praveen, Mallepu S. Likhitha Reddy, Rahul Haribhau Wadghane, Atul Kumar Shrivastava, Manish Patel

**Affiliations:** 1 Department of Farm Machinery and Power Engineering, College of Agricultural Engineering, Jawaharlal Nehru Krishi Vishwa Vidyalaya, Jabalpur, Madhya Pradesh 482004 India; 2 Department of Agronomy, SR University, Warangal, Telangana 506371 India; 3 Symbiosis Institute of Operations Management, Symbiosis International (Deemed University), Nashik Campus, Pune, Nasik, Maharashtra 422008 India

**Keywords:** Fenugreek, Battery-operated harvester, Agricultural mechanization, Leafy vegetable harvesting, Harvesting efficiency, Cutting efficiency, Engineering, Plant sciences

## Abstract

Manual harvesting of fenugreek (*Trigonella foenum-graecum* L.) leaves using sickles requires intensive labor and forces workers to adopt prolonged stooped postures, often resulting in musculoskeletal strain and reduced operational efficiency. To address these limitations, a self-propelled battery-operated leafy vegetable harvester was developed and evaluated under field conditions in fenugreek. Machine performance was assessed using a factorial experimental design with three forward speeds (1.5, 2.0, and 2.5 km h^−1^), three conveyor driving pulley diameters (50.8, 76.2, and 101.6 mm), and two cutting heights (50 and 75 mm). The developed prototype achieved a theoretical field capacity of 0.16 ha h^−1^ and an effective field capacity of 0.13 ha h^−1^, resulting in a field efficiency of 81.25%. The machine operated continuously for 6.5 h on a single battery charge. Performance evaluation indicated a cutting efficiency of 98.1%, harvesting efficiency of 97.15%, and a conveying loss of 0.95%. Under a cut-and-come-again harvesting system, agronomic yields were 30.7 q ha^−1^ at 31 days after sowing (DAS), followed by 24.2 q ha^−1^ and 20.3 q ha^−1^ at 47 and 61 DAS, respectively. These results demonstrate that the developed battery-operated harvester provides an efficient mechanized solution for fenugreek harvesting, reducing labor drudgery while improving harvesting efficiency and operational productivity. The study highlights the novelty of integrating crop-specific mechanical design with optimized operational parameters to enable efficient mechanized harvesting of fenugreek under a cut-and-come-again production system.

## Introduction

Early research on sustainability defines progress in social, environmental, and economic areas as the core goal of sustainable development^1^. Even in countries with adequate financial resources and relatively stronger governance systems, there is still a lack of clear strategies to properly integrate the social, economic, and environmental components in order to achieve sustainable agricultural practices^2^. This challenge brings into focus the importance of comprehensive approaches that stablish the triple-dimensions of the concept of sustainability at the same time in order to facilitate a more sustainable agricultural development over time^3^. Sustainable agriculture advocates for agricultural technology and farming practices that will secure the long-term viability of the country’s agricultural resources while ensuring environmental responsibility and the role of farmers^4^. The use of advanced technological innovations in agriculture plays an important role in improving productivity and operational efficiency, closing the labour shortage, lowering the cost of production, improving product quality, and making agriculture sustainable^5,6^.

Agricultural mechanization has brought about significant changes in modern agricultural operations by replacing manual labour with sophisticated technological solutions that have improved the efficiency of agricultural tasks such as planting, harvesting, crop handling, and worker safety^7^. However, traditional agricultural machinery (i.e. tractors, combines and harvesters) requires a lot of fossil fuels and therefore it raises the cost of production and adds to environmental pollution^8,9^. With growing global concern about environmental sustainability, many countries are promoting the shift to green energy technologies to minimize the reliance on fossil fuels^10^. Electrified agricultural equipment provides a number of benefits, including lower operating costs, lower noise levels and reduced emission of greenhouse gases, hence providing environmentally sustainable alternatives for agricultural operations^11^. Therefore, more studies and technology development are necessary to design agricultural machinery with low energy consumption and less impact on the environment to promote a sustainable agricultural system^12^.

Leafy vegetables are cultivated extensively throughout the world owing to their high nutritional and medicinal value. India has a rich biodiversity of plants, with around one-third of its 6,000 plant species being edible green leafy vegetables. In the last years, the consumption of green leafy vegetables as functional food that provides a flavour, aroma and nutritional values is grown considerably^13^.

Among these crops, fenugreek (*Trigonella foenum-graecum* L.) is among the oldest ones and cultivated in most parts of the world as one of the most widely consumed leafy vegetables (Tania et al., 2024). Fenugreek leaves are regarded as an important part of the diet because of its high level of proteins, fibre, vitamins, and minerals^14^. They are also economical and widely used in traditional and modern day food systems^15,16^. In addition, fenugreek extracts have been shown to have a number of physiological and pharmacological benefits in both human and animal studies^17,18^.

Despite the importance of the food, farming of fenugreek is still done mostly by hand, using sickles. During the harvesting, workers are forced to work in a sitting or bending posture, these results in physical strain, fatigue, and low harvesting efficiency. Harvesting fenugreek by cutting as opposed to uprooting has a number of agronomic advantages. In the cut-and-come-again method of harvesting crops, the farmers are able to get fresh supply of leafy vegetables over a long period of time, after a single sowing operation. This system is especially beneficial for smallholder farmers where the grain can be harvested multiple times in a period of ~ 6–7 months, thus giving them a sustained source of income with extremely high yield of leafy vegetation and seeds^19,20^. Furthermore, crop stubble maintenance contributes to a decrease of soil erosion, a decrease of moisture loss by lowering the wind velocity on the soil surface, a reduction of raindrop impact and an enhancement of soil moisture retention by a reduction of soil run-off^21,22^.

Although reciprocating cutter bars and conveyor-based machines are used widely in the harvesting equipment, in most of the small-scale leafy vegetable harvesters the design is generalized without crop-specific mechanical programmer and without experimentally validated optimization of the parameters. Machine performance during harvesting operations is influenced to a large extent by operational parameters such as forward speed, cutting height and conveyor speed which has a direct effect on cutting efficiency, conveying losses and harvesting performance. However, limited research has been done on optimizing these operational parameters for use with fenugreek harvesting under cut and come again production systems.

In order to fill this gap in the available research, the current study combines the morphology of the fenugreek crop with synchronous mechanical operation that is appropriate for regenerative harvesting conditions. Cutting heights of 50 mm and 75 mm were chosen using the characteristics of basal stems and regenerative nodes locations to ensure active growing points remain for the following sowing harvest cycle. From the mechanical point of view, the developed system includes controlled kinematic synchronization of forward velocity (V_f_), reel peripheral speed (V_r_) and conveyor belt velocity (V_c_) via selectable pulley configurations. This synchronisation controls the flow of biomass at the cutter-reel-conveyor interface, which is beneficial to reduce crop accumulation, minimise the conveying loss and stabilise the harvesting efficiency at different operational speeds.

In addition to the mechanical design, a three factor factorial experiment that was set in a randomized block design was used to test the individual and combinations of the forward speed, the pulley diameter and cutting height on the harvesting performance of the developed machine. Statistical modelling and response-based optimization with the design expert software application were used to optimize the operational parameter combinations from which the maximum efficiency of harvesting coupled with the minimum loss in conveying can be achieved. Therefore, the aim of this research was to design a crop specific self-propelled battery operated harvester for fenugreek and also to experimentally study the effects of various forward speed, pulley diameter and cutting heights on the machine performance under regenerative cut and come-again harvesting condition.

## Materials and methods

### Description of the developed self-propelled battery-operated Fenugreek harvester

#### Working principle of the harvester

**Fig. 1:**
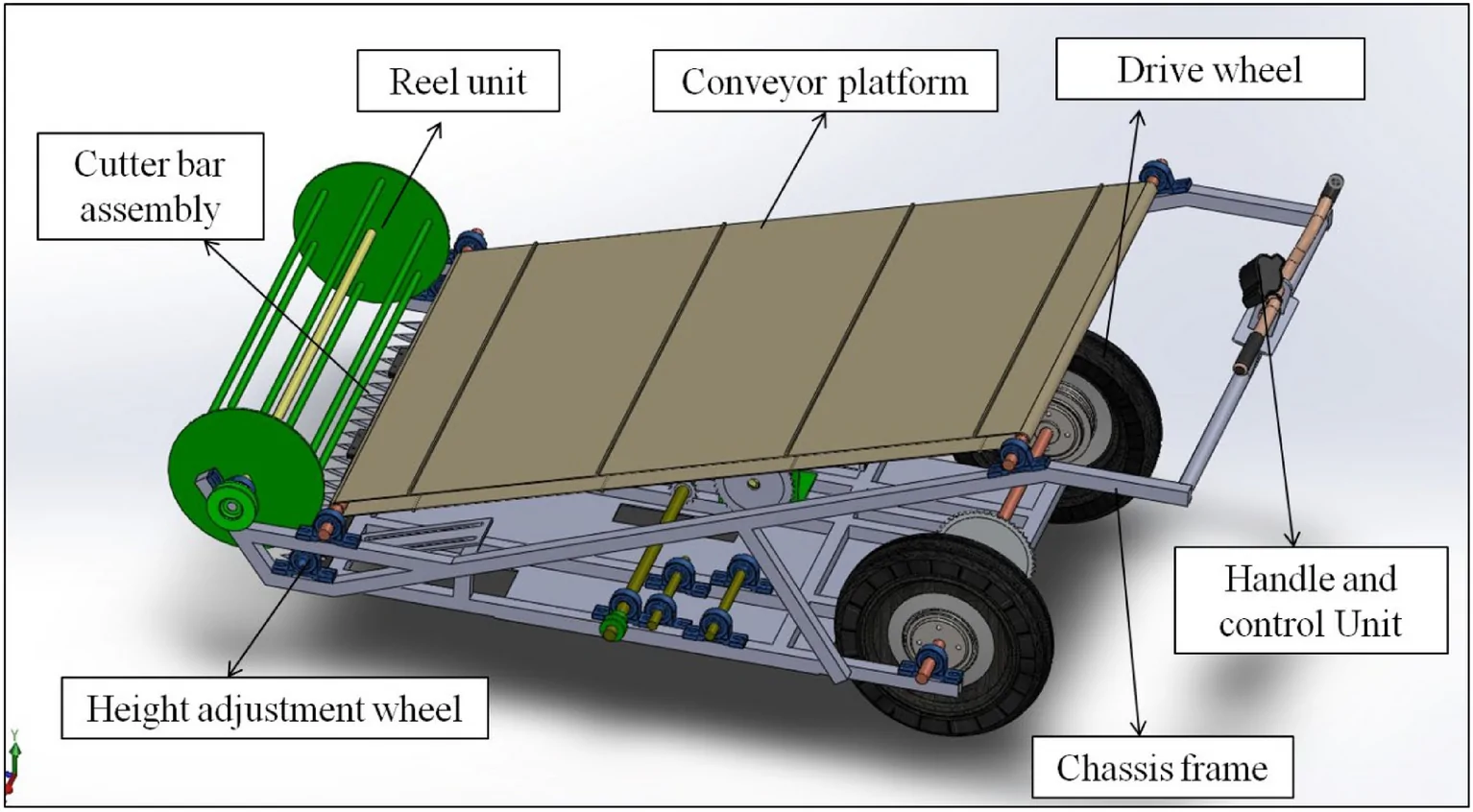
Overview of the developed self-propelled battery-operated fenugreek harvester showing the structural frame, cutter bar assembly, conveyor platform, reel unit, and drive mechanism.

The developed Self-propelled Battery-operated Fenugreek Harvester (Fig. 1) is aimed to increase the crop harvesting efficiency in fenugreek crop harvesting by automating the cutting and gathering process, and hence reduce the manual labour and to streamline the harvesting process. The machine is powered by an integrated rechargeable battery system, thus removing the need for conventional fuel or power sources, and offers a sustainable energy solution for the motor and ancillary electrical parts.

Operation is activated by switching the starting switch to the right position and starting the operation of the Main Circuit Breaker (MCB), at which time the digital display screen shows the current battery voltage level. Engaging the throttle activates all the machinery’s working units, such as the cutting mechanism, rear wheels, conveyor system, reel unit, and auxiliary parts. As the harvester passes across the field, the cutting device makes a controlled cutting at the basal portion of the fenugreek plants. Cut material is then transferred by the conveyor system consisting of a series of belts or rollers from the cutting zone to the desired collection unit. The operator has control of forward motion and speed through the throttle allowing for smooth and responsive operation on varying field terrain.

The cutter bar was mounted on a carriage assembly incorporated in the wheel supporting structure on the front. Vertical positioning of the cutter unit relative to the ground surface was controlled by a slot-based frame adjustment mechanism that allowed maintaining predefined cutting heights during field operation. On the front wheels the ground contact was stable and the clearance was consistent between the cutter bar and the soil surface. Technical specifications of developed harvester are shown in Table 1.

**Table 1:** Technical specifications of the developed self propelled battery operated fenugreek harvester.

Parameter	Specification
Machine type	Self-propelled battery-operated leafy vegetable harvester
Target crop (paper focus)	Fenugreek (cut-and-come-again system)
Cutting mechanism	Serrated reciprocating cutter bar
Cutting width	400 mm
Blade thickness	2 mm
Pitch of serrated knives	12 mm
Cutting height levels tested	50 mm and 75 mm
Forward speed levels tested	1.5, 2.0 and 2.5 km h^−1^
Conveyor type	Belt conveyor with cleats
Conveyor width	300 mm
Conveyor length	1400 mm
BLDC motor	Brushless DC motor
Battery type	Lead-acid battery
Battery rating	48 V system
Transmission system	Belt–pulley and chain–sprocket drive
Reel unit	Power-driven guiding reel
Reel Speed Index (RSI)	Ratio of reel peripheral speed to forward speed

#### Design basis and constructional considerations

The design of the developed fenugreek harvester was based on experimentally determined agronomical, physical and mechanical properties of fenugreek plants measured prior to fabrication. These parameters included plant height, stem diameter, crop stand density, biomass yield per unit area and stem cutting resistance, and were used to determine the design requirements of the cutting, conveying, and propulsion subsystems. A serrated reciprocating cutter bar was used after measured stem characteristics. A blades thickness of 2 mm was selected in order to have adequate rigidity in the structure and also to reduce cutting resistance. The serration pitch of 12 mm was chosen to increase the shearing mechanism and diminish the required force to sever at the base, which in turn ensures clean cutting with minimum tissue damage.

The cutting width of 400 mm was obtained based on the density of the crop stand and allowable draft for stable operation in small plot conditions. Cutting heights of 50 mm and 75 mm were taken into consideration of the regenerative growth nature of fenugreek using the cut and come again harvesting system. These levels guaranteed maintenance of basal regenerative nodes while allowing for good the recovery of biomass. The cutter unit was installed on a vertically adjustable carriage frame carried on wheels that made contact with the ground, allowing the cutting height to be kept uniform, eliminating the variation during cutting. This arrangement provided also the minimal soil contamination and reduced cutting resistance in lower stem regions. The power requirement of the machine was estimated according to torque and rotational speed requirements in maximum operating conditions (2.5 km h^−1^). The total power demand was used to select a 48 V BLDC motor with sufficient torque capacity and operational safety margin which can ensure stable performance under peak biomass load conditions.

#### Estimation of power requirement

Power of individual subsystem was calculated according to torque requirement and rotational speed at maximum forward speed conditions (2.5 km h^−1^). The total load calculated was used to choose a BLDC motor of suitable 48 V with an adequate turbine torque capacity and safety margin operational margin to ensure a stable motor operation in the absence of overloads in the maximum biomass operation conditions. The power requirement of the developed harvester was determined by the estimation of torque and rotational speed requirements of individual subsystems under maximum operational load.

Power for cutter unit (P_c_) was calculated as:

$${\mathrm{P}}_{{\mathrm{c}}} = \frac{{{\mathrm{F}}_{{\mathrm{c}}} \times {\mathrm{v}}_{{\mathrm{c}}} }}{{\eta _{{\mathrm{c}}} }}$$

where,

F_c_ = cutting force (N) determined from measured stem cutting resistance of fenugreek,

v_c_ = cutter bar linear velocity (m s^−1^),

η_c_ = mechanical efficiency of cutter drive system.

Power for reel unit (Pr) was estimated from torque required to lift and guide crop biomass:

$${\mathrm{P}}_{{\mathrm{r}}} = \frac{{{\mathrm{T}}_{{\mathrm{r}}} \times \omega _{{\mathrm{r}}} }}{{\eta _{{\mathrm{r}}} }}$$

where,

T_r_ = torque on reel shaft (N·m),

ω_r_ = angular velocity of reel (rad s^−1^),

η_r_ ​ = transmission efficiency.

Power for conveyor system (P_conv_) was calculated based on biomass load and belt velocity:

$${\mathrm{P}}_{{{\mathrm{conv}}}} \frac{{{\mathrm{F}}_{{{\mathrm{belt}}}} \times {\mathrm{v}}_{{{\mathrm{belt}}}} }}{{\eta _{{{\mathrm{conv}}}} }}$$

where,

F_belt_​ = force required to move cut biomass on conveyor (N),

v_belt_ = conveyor belt velocity (m s^−1^).

Power for propulsion system (Pw) was estimated from draft requirement and forward velocity:

$${\mathrm{P}}_{{\mathrm{w}}} = \frac{{{\mathrm{F}}_{{\mathrm{d}}} \times {\mathrm{V}}_{{\mathrm{f}}} }}{{\eta _{{\mathrm{w}}} }}$$

where,

F_d_ = draft force (N),

V_f_​ = forward speed (m s^−1^).

The total power requirement was determined as:

$${\mathrm{P}}_{{{\mathrm{total}}}} {\text{ = P}}_{{\mathrm{c}}} {\text{ + P}}_{{\mathrm{r}}} {\text{ + P}}_{{{\mathrm{conv}}}} {\text{ + P}}_{{\mathrm{w}}}$$

The total power requirement of the system was determined as the sum of all subsystem power requirements. The calculated maximum power demand under 2.5 km h^−1^ was used as the basis for selecting a 48 V BLDC motor.

#### Selection of pulley diameters and transmission system

Pulley diameters were determined based on transmission ratio requirements necessary to regulate the rotational speeds of the reel and conveyor subsystems relative to the forward velocity of the harvester. In a belt pulley transmission system, the angular velocity relationship between the driving and driven pulleys is governed by:

$${\mathrm{N}}_{1} {\mathrm{D}}_{1} = {\mathrm{N}}_{2} {\mathrm{D}}_{2}$$

where,

N_1_, N_2_ = rotational speeds (rev min^−1^).

D_1_, D_2_ = pulley diameters (m).

Variation in diameter of pulley directly modifies transmission ratio and thus changes the angle velocity of driven shaft. This change has implications for both reel peripheral speed and for conveyor belt velocity which are important factors in controlling dynamics of crop lifting and biomass transfer. The chosen pulley diameters (50.8 mm, 76.2 mm and 101.6 mm) were chosen to achieve three discrete transmission ratios, which could be used to achieve measurable variation in reel speed index (RSI) and conveyor velocity for the tested forward speeds (1.5, 2.0 and 2.5 km h^−1^). The selected range ensured the existence of good differentiation in kinematic behavior within the limits of torque and power of the 48 V BLDC motor at maximum load conditions. This method of structured selection allowed the systematic investigation of the kinematic synchronization between forward movement, reel movement, and conveyor moving under the framework of this factorial experimental design.

#### Blade soil interface adjustment

**Fig. 2:**
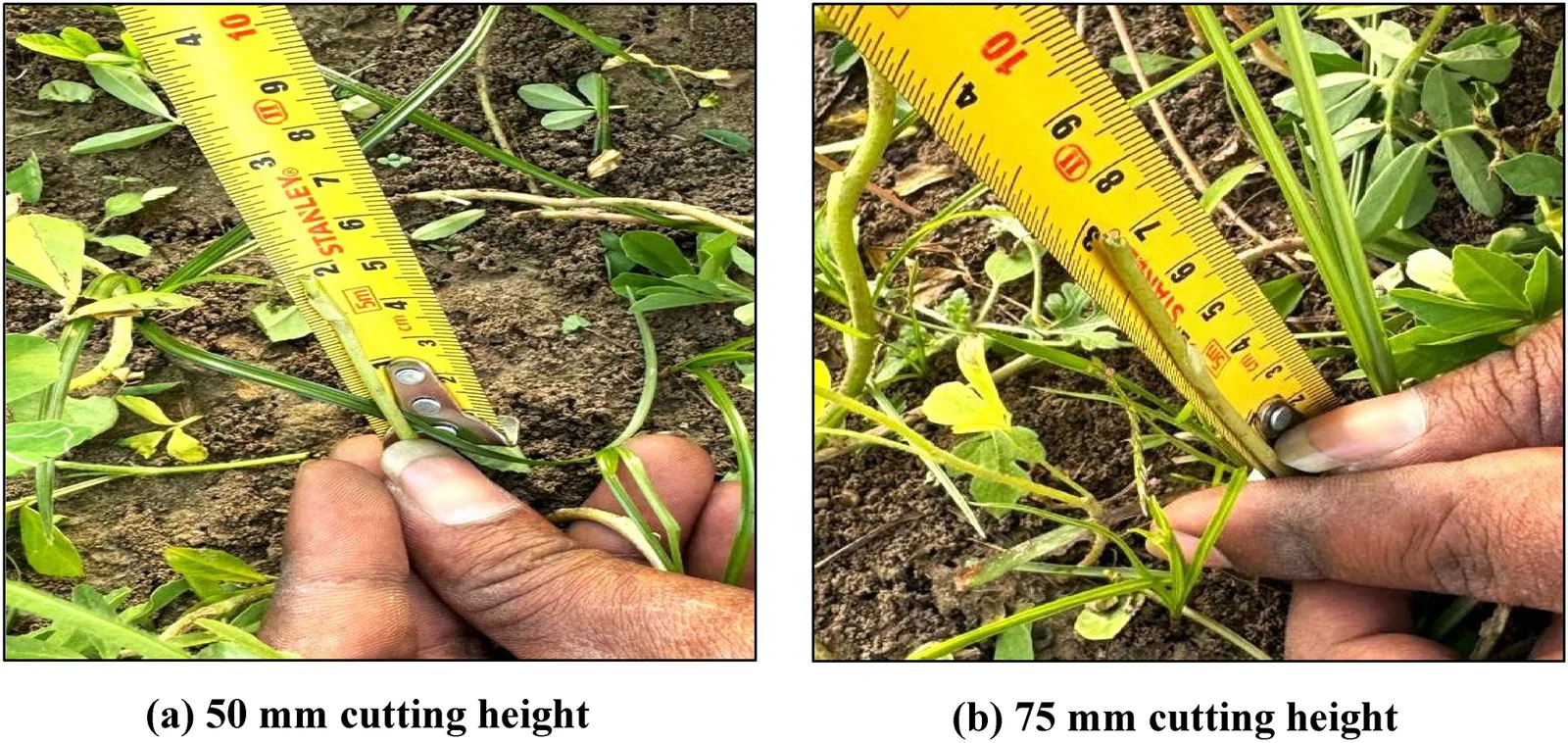
Effect of cutting height on fenugreek harvesting: (**a**) 50 mm cutting height and (**b**) 75 mm cutting height.

To assure uniformity of blade elevation to the surface of the soil during field operation, the cutter bar assembly was mounted on a vertically adjustable carriage frame incorporating slotted supports for height calibration. The required cutting height (50 mm or 75 mm) was set before experimentation and fixed using mechanical locking bolts to avoid the risk of movement during dynamic loading conditions. Two cutting height levels (50 mm and 75 mm) were chosen in order to assess the compromise between biomass recovery and growth potential (Fig. 2).

Front ground support wheels were structurally aligned with the cutter assembly in order to maintain consistent contact with the ground and also to stabilize the blade soil interface during forward progression. This configuration reduced vertical oscillation of the cutter unit and also ensured uniform cutting height across experimental plots. The combined slot-adjustment and wheel support mechanism ensured controlled and repeatable blade soil clearance throughout all combinations of treatment.

#### Kinematic synchronization of forward speed, conveyor speed and reel speed

The performance of the developed self-propelled battery-operated fenugreek harvester depends on proper synchronization between forward speed (V_f_), conveyor belt velocity (V_c_), and reel peripheral speed (V_r_), which collectively govern cutting, biomass transfer, and overall harvesting efficiency.

The conveyor belt velocity was determined based on the diameter and rotational speed of the conveyor drive pulley and was expressed as:

$${\mathrm{V}}_{{\mathrm{c}}} = \, \pi {\mathrm{DN}}$$

where,

V_c_ = conveyor velocity (m s^−1^),

D = pulley diameter (m), and.

N = rotational speed (rev s^−1^).

For efficient biomass transfer, the conveyor velocity was maintained equal to or slightly greater than the forward speed (Vc ≥ Vf). If Vc < Vf, biomass accumulation occurs ahead of the cutter bar, increasing conveying loss, whereas excessive conveyor velocity may lead to unstable crop handling and energy inefficiency. The forward speed was evaluated at three levels of 1.5, 2.0, and 2.5 km h^−1^.

The reel peripheral speed was calculated as:

$${\mathrm{V}}_{r} = \pi {\mathrm{D}}_{r} {\mathrm{N}}_{r}$$

where,

V_r_ = reel peripheral speed (m s^−1^),

D_r_ = reel diameter (m), and.

N_r_ = reel rotational speed (rev s^−1^).

The reel facilitates proper lifting and guiding of crop towards the cutter bar. The synchronization between reel motion and forward speed was expressed using the reel speed index (RSI):

$$\mathrm{R}\mathrm{S}\mathrm{I}= \frac{{\mathrm{V}}_{\mathrm{r}}}{{\mathrm{V}}_{\mathrm{f}}}$$

Appropriate RSI values ensured efficient crop feeding without excessive pushing or dragging. Overall, synchronization among V_f_, V_c_ and V_r_ enabled smooth crop flow through the cutter – reel – conveyor system, minimizing biomass accumulation and conveying loss while improving harvesting efficiency.

### Experimental site and field conditions

The field experiment was conducted at Research Farm of College of Agricultural Engineering, Jawaharlal Nehru Krishi Vishwa Vidyalaya (JNKVV) Jabalpur, Madhya Pradesh, India at typical field conditions suitable for fenugreek cultivation. The crop was developed under a cut and come again harvesting system in order to facilitate multiple harvesting on a single sowing while leaving regenerative growth nodes intactExperimental layout and the variables considered for field evaluation of developed harvester are presented in Table 2. The study was carried out using fenugreek as the test crop and the performance of the machine was tested by varying the key operational parameters such as the conveyor driving pulley diameter, forward speed, and cutting height. The driving pulley diameter on the conveyor was studied at three levels i.e. 50.8 mm, 76.2 mm and 101.6 mm, while the forward speed was studied at 1.5, 2.0 and 2.5 km h^−1^ along with the cutting height at 50 mm and 75 mm.

**Table 2:** Experimental layout of the harvester field trial.

S. no	Independent variable		Dependent variables
1	Crop	Fenugreek	1. Effective field capacity, (ha/h) 2. Theoretical field capacity, (ha/h) 3. Field efficiency, (%) 4. Cutting efficiency, (%) 5. Conveying loss, (%) 6. Harvesting efficiency, (%)
2	Machine level (3)	Conveyor driving pulley; M_1_ = 50.8 mm pulley, M_2_ = 76.2 mm pulley, M_3_ = 101.6 mm pulley	
3	Forward Speed (3)	1.5 km h^−1^, 2 km h^−1^, 2.5 km h^−1^	
4	Cutting height (2)	50 mm, 75 mm	

The dependence performance parameters determined in the experiment were the effective field capacity, theoretical field capacity, field efficiency, cutting efficiency, conveying loss and harvesting efficiency as summarised in Table 2. These parameters were chosen for the purpose of a thorough evaluation of the functional performance of the developed harvester under different conditions of operation. Before harvesting the physical properties of the soil were determined to describe the field situations. Soil samples were taken from different places in the experimental area and parameters such soil moisture content, bulk density, and cone index were measured by standard procedures^23–25^. The mean soil moisture content was 19.31% (dry basis), bulk density was 1.23 g cm^−3^, and cone index at 100 mm depth was 13.12 kg cm^−2^ which shows appropriate field conditions for machines operation. All the experimental plots were under consistent agronomic and irrigation practices so that external variability was minimized. Observations were taken on each experimental run for all of the performance parameters under actual field operating conditions for each of the treatment combinations.

### Experimental design and Statistical analysis

The performance evaluation of developed fenugreek harvester was conducted using three factor factorial experiment design in Randomized Block Design (RBD) under field conditions to study individual and interaction effects of operational parameters. The forward speed, conveyor driving pulley diameter, and cutting height were the independent variables. Forward speed was tested at three levels (1.5, 2.0 and 2.5 km h^−1^), pulley diameter at three levels (50.8, 76.2 and 101.6 mm) and cutting height at two levels (50 mm and 75 mm). The experimental factors, their symbols and corresponding levels are summarized in Table 3 in a structured representation of the factorial design. Cutting height was included as one of the critical independent parameters because it also affects harvesting efficiency, biomass recovery, stem cutting resistance and regenerative capacity of fenugreek under a cut-and-come-again system. Fenugreek does regenerate via basal axillary nodes above the soil surface which are concentrated in a defined basal zone so as the cutting height affects the regrowth potential that is preserved. From the mechanical point of view the rigidity of stem and cutting resistance increases from one end (base) toward the other, due to higher tissue density. As a result lower cutting heights have higher cutting force requirement while higher cutting heights have less resistance and potentially lower biomass recovery. Accordingly, implemented cutting height concurrently controls biological regeneration and mechanical cutting and two levels (50 mm and 75 mm) were chosen to assess this trade-off.

**Table 3:** Experimental factors and levels used in the factorial design for evaluation of the fenugreek harvester.

Factor	Symbol	Levels
Forward speed (km h^−1^)	V_f_	1.5, 2.0, 2.5
Pulley diameter (mm)	D_p_	50.8, 76.2, 101.6
Cutting height (mm)	H_c_	50, 75

The factorial combination of the variables led to a total of 18 treatment combinations (3 × 3 × 2), replicated three times for a total of 54 experimental observations. A RBD was used to reduce variability within the field and maximize the precision of the treatments, in that treatments were randomly allocated within each replication. The experimental design allowed for the evaluation of main effects and interaction effects between forward speed, pulley diameter and cutting height. Statistical analysis was done using Design-Expert software and analysis of variance (ANOVA) analysis was done to determine the significance of effect of the operational parameters. Model adequacy was evaluated in terms of F-values, *P*-values, R^2^, adjusted R^2^, predicted R^2^, and lack of fit tests with a level of significance of *p* < 0.05. Response surface methodology (RSM) was used for the analysis of variable interaction as well as finding predictive models for cutting efficiency, conveying loss and harvesting efficiency. Optimization of the operational parameters was conducted by using desirability function method to maximise cutting and harvesting efficiency while harvesting conveying loss.

### Soil physical properties

The physical properties of the soil at the experimental site were determined prior to conducting the harvesting trials to characterize the field conditions under which the developed harvester was evaluated. Soil samples were collected systematically from different locations within the experimental area to obtain representative measurements.

Soil moisture content was determined using the standard oven-drying method, wherein soil samples were weighed, dried at a controlled temperature, and reweighed to compute moisture content on a dry basis. Bulk density of the soil was measured using the core cutter method, which involves collecting undisturbed soil samples of known volume and determining their mass after drying. The cone index, representing soil penetration resistance, was measured using a cone penetrometer at a depth of 100 mm. All measurements were conducted using standard procedures as described by^23–25^. These parameters were essential for assessing soil conditions affecting machine mobility, cutting stability, and overall field performance.

### Field performance evaluation

**Fig. 3:**
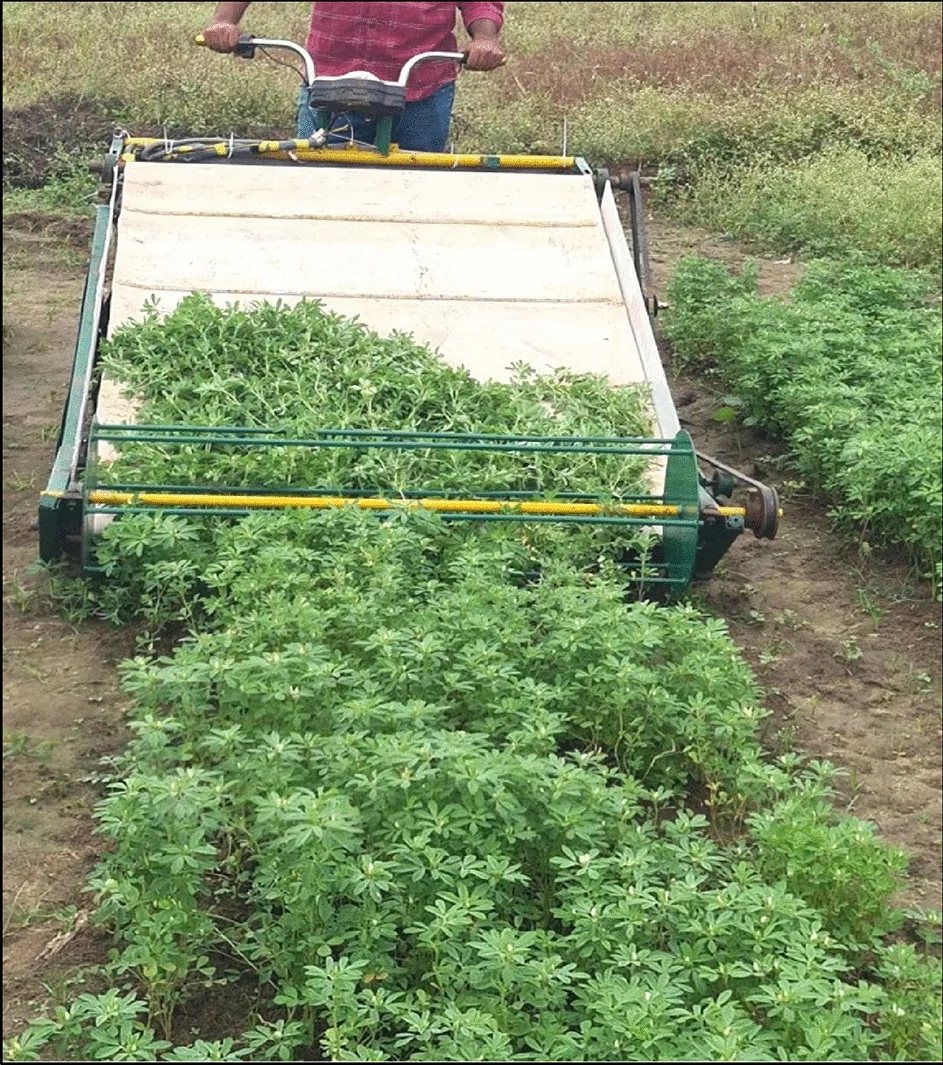
Field evaluation of the developed fenugreek harvester under actual operating conditions during harvesting.

The performance of the developed fenugreek harvester was evaluated under field conditions by measuring key operational parameters related to machine efficiency, crop handling, and field capacity. The developed harvester was evaluated under field conditions to assess its operational performance (Fig. 3). The evaluation was conducted for each treatment combination, and observations were recorded systematically.

#### Theoretical field capacity

The theoretical field capacity of the harvester was calculated based on the effective width of operation and forward speed using the standard relationship^25^.

$$\mathrm{T}.\mathrm{F}.\mathrm{C}=\frac{\mathrm{S}\times \mathrm{W}}{10}$$

where,

S = forward speed (km h^−1^).

W = Effective cutting width (m).

#### Effective field capacity

During the test, the whole harvesting time of the harvester was taken, including the time spent on activities such as turning and emptying the yield collecting bag. To assess the machine’s efficiency, the effective field capacity was then calculated based on these recorded times^25^.

$$\mathrm{E}.\mathrm{F}.\mathrm{C} (\mathrm{h}\mathrm{a}/\mathrm{h})=\frac{\mathrm{A}}{{\mathrm{T}}_{\mathrm{p}}+{\mathrm{T}}_{\mathrm{n}}}$$

where,

A = area harvested (ha).

T_p_ = productive time (h).

T_n_​ = non-productive time (h).

#### Field efficiency

The field efficiency was calculated using the following equation^25^.

$${\mathrm{Field}}\;{\mathrm{efficiency}} = \frac{{{\mathrm{E}}.{\mathrm{F}}.{\mathrm{C}}}}{{{\mathrm{T}}.{\mathrm{F}}.{\mathrm{C}}}} \times 100$$

#### Cutting efficiency

The harvester’s cutting efficiency (CE) was calculated by dividing the total number of cut plants (W_1_) by the total number of plants (W_2_) present prior to the plot’s harvesting process. The average cutting efficiency was obtained using three replications^26^.

$${\mathrm{CE}} = \frac{{W1}}{{W2}} \times 100$$

Where,

W_1_ = number of plants cut by machine.

W_2_ = total plants present before harvest.

#### Conveying loss

Conveying loss is the harvested crop left in the field after machine operation. It was determined by using equation

$$\mathrm{C}\mathrm{o}\mathrm{n}\mathrm{v}\mathrm{e}\mathrm{y}\mathrm{i}\mathrm{n}\mathrm{g}\, \mathrm{l}\mathrm{o}\mathrm{s}\mathrm{s} \left(\mathrm{\%}\right)=\frac{{W}_{l}}{{W}_{t}} \times 100$$

where,

W_l_ = weight of crop dropped during conveying (kg).

W_t_ = total harvested crop entering the conveyor (kg).

#### Harvesting efficiency

Harvesting efficiency represents the percentage of crop successfully harvested by the machine after accounting for cutting and conveying losses. It was calculated using the following relationship.

$$\mathrm{H}\mathrm{a}\mathrm{r}\mathrm{v}\mathrm{e}\mathrm{s}\mathrm{t}\mathrm{i}\mathrm{n}\mathrm{g}\, \mathrm{E}\mathrm{f}\mathrm{f}\mathrm{i}\mathrm{c}\mathrm{i}\mathrm{e}\mathrm{n}\mathrm{c}\mathrm{y} \left(\mathrm{\%}\right)= \frac{{\mathrm{W}}_{\mathrm{h}}}{{\mathrm{W}}_{\mathrm{t}}} \times 100$$

where.

W_h_ = weight of crop successfully harvested.

W_t_ = total crop available in the sampling area.

#### Crop sampling and measurement procedure

For determining cutting efficiency and conveying loss, crop sampling was carried out using a 1 m^2^ quadrat placed within the harvested experimental plots during machine operation. Before harvesting, the quadrat was randomly positioned in the test plot and the total number of plants present within the 1 m^2^ area was counted to determine the initial plant population (W₂). After the developed harvester passed through the same section, the number of plants successfully cut within the quadrat was counted to determine the harvested plants (W₁), and the number of plants left uncut was also recorded. Crop material dropped during the conveying process and crop left on the ground were carefully collected from the same 1 m^2^ sampling area and weighed separately using a digital weighing balance to estimate conveying loss. The developed harvester had an effective cutting width of 400 mm (0.40 m). Each treatment combination was evaluated under five replications, and the average values obtained from these observations were used to calculate cutting efficiency, conveying loss, and harvesting efficiency of the developed fenugreek harvester.

### Economic analysis

The economic feasibility of the developed battery-operated self-propelled leafy vegetable harvester was evaluated by estimating the operational cost of the machine and comparing it with conventional manual harvesting practices. The total development cost of the harvester was ₹61,908. The economic life of the machine was assumed to be 8 years with an average annual use of 960 h per year, excluding approximately four rainy months. An annual interest rate of 10% was considered for cost calculations. The economic performance of the developed battery-operated fenugreek harvester was evaluated using standard farm machinery cost analysis procedures. The assumptions used for estimating the fixed and operating costs of the machine are presented in Table 4.

**Table 4:** Assumptions used for economic evaluation of the developed battery-operated fenugreek harvester.

Parameter	Value	Unit
Initial cost of harvester	61,908	₹
Expected life of machine	8	years
Average annual use of machine	960	h year^−1^
Interest rate	10	%
Repair and maintenance cost	10	% of machine cost
Electricity cost for battery charging	0.588	₹ h^−1^
Operator wage rate	300	₹ day^−1^
Labour requirement	2	persons
Salvage value (assumed)	10	% of initial cost

The cost of operation of the machine was estimated by determining the fixed costs and variable costs following standard agricultural machinery cost analysis procedures.

The fixed cost of the machine was calculated by considering depreciation, interest on investment, and housing costs. Depreciation was determined using the straight-line method as:

$$D= \frac{C-S}{L \times H}$$

where,

D is depreciation cost (₹ h^−1^),

C is the initial cost of the machine (₹),

S is the salvage value (₹),

L is the economic life of the machine (years), and.

H is the annual working hours of the machine (h).

Interest on investment was calculated using:

$$I= \frac{(C+S)}{2}\times \frac{i}{H}$$

where,

I is the interest cost (₹ h^−1^) and.

i is the annual interest rate.

Variable costs included repair and maintenance cost, electricity cost for battery charging, and labor wages. Repair and maintenance cost was estimated as a percentage of the machine cost, electricity cost was determined based on the energy consumption during battery charging, and labor cost was calculated based on the daily wage rate of the operator and assisting labor required for machine operation.

The total cost of operation of the harvester was calculated as:

$$\mathrm{T}\mathrm{C}=\mathrm{F}\mathrm{C}+\mathrm{V}\mathrm{C}$$

where,

TC is the total operating cost (₹ h^−1^),

FC is the fixed cost (₹ h^−1^), and.

VC is the variable cost (₹ h^−1^).

The cost of harvesting per hectare was estimated using the effective field capacity of the machine:

$$\mathrm{C}\mathrm{H}=\mathrm{T}\mathrm{C} \times \mathrm{T}$$

where,

CH is the harvesting cost (₹ ha^−1^) and.

T is the time required to harvest one hectare.

#### Break-even point

The break-even point represents the minimum annual utilization of the machine at which the total revenue generated from custom hiring equals the total cost of ownership and operation. At this point, the machine neither generates profit nor incurs loss. The break-even point of the developed fenugreek harvester was calculated using the following equation:

$$\mathrm{B}\mathrm{E}\mathrm{P}= \frac{\mathrm{A}\mathrm{F}\mathrm{C}}{\mathrm{C}\mathrm{H}-\mathrm{C}}$$

where,

BEP = break-even point (h year^−1^).

AFC = annual fixed cost of the machine (₹ year^−1^).

CH = custom hiring charge of the machine (₹ h^−1^).

C = operating cost of the machine (₹ h^−1^).

#### Payback period

The payback period indicates the time required to recover the initial investment made for the development of the machine through the annual net benefits obtained from its operation.

$$\mathrm{P}\mathrm{a}\mathrm{y}\,\mathrm{b}\mathrm{a}\mathrm{c}\mathrm{k}\, \mathrm{p}\mathrm{e}\mathrm{r}\mathrm{i}\mathrm{o}\mathrm{d}\,= \frac{\mathrm{I}\mathrm{n}\mathrm{i}\mathrm{t}\mathrm{i}\mathrm{a}\mathrm{l}\, \mathrm{i}\mathrm{n}\mathrm{v}\mathrm{e}\mathrm{s}\mathrm{t}\mathrm{m}\mathrm{e}\mathrm{n}\mathrm{t}}{\mathrm{A}\mathrm{n}\mathrm{n}\mathrm{u}\mathrm{a}\mathrm{l}\, \mathrm{n}\mathrm{e}\mathrm{t}\, \mathrm{b}\mathrm{e}\mathrm{n}\mathrm{e}\mathrm{f}\mathrm{i}\mathrm{t}}$$

where,

Initial investment = total cost of machine (₹).

Annual net benefit = annual income obtained from machine operation (₹ year^−1^).

Annual net benefit was calculated as:

$$\mathrm{A}\mathrm{n}\mathrm{n}\mathrm{u}\mathrm{a}\mathrm{l}\, \mathrm{n}\mathrm{e}\mathrm{t}\, \mathrm{b}\mathrm{e}\mathrm{n}\mathrm{e}\mathrm{f}\mathrm{i}\mathrm{t}\,=\left(\mathrm{C}\mathrm{H}-\mathrm{C}\right)\times \mathrm{A}\mathrm{U}$$

where,

CH = custom hiring charge (₹ h^−1^).

C = operating cost of machine (₹ h^−1^).

AU = average annual use of machine (h year^−1^).

#### Benefit – cost ratio

The benefit–cost ratio (B:C ratio) is used to evaluate the economic viability of the developed harvester by comparing the benefits obtained from its use with the cost of operation.

$${\mathrm{B}}:{\mathrm{C}} = \frac{{{\mathrm{Annual}}{\mkern 1mu} {\mathrm{net}}{\mkern 1mu} {\mathrm{benefit}}}}{{{\mathrm{Cost}}\;{\mathrm{of}}\;{\mathrm{operation}}{\mkern 1mu} }}$$

where,

Annual net benefit = annual profit obtained from machine operation (₹ year^−1^).

Cost of operation = cost incurred in harvesting per hectare (₹ ha^−1^).

## Results and discussion

### Field condition before harvesting

Field conditions have an important role to play in determining operational performance of harvesting machinery. Therefore, key soil physical properties of the experimental field were measured before conducting the harvesting trials. The mean soil moisture content was found to be 19.31% (dry basis) and mean soil bulk density was 1.23 g cm^−3^ while the mean cone index was evaluated at a depth of 100 mm and was found to be 13.12 kg cm^−2^. These parameters were found according to the standard measurement procedures for soils^24,25^. The observed soil conditions were regarded as suitable for the evaluation of field performance of the developed fenugreek harvester.

### Crop parameters before harvesting

Crop characteristics are known to play an important part in harvesting machinery performance, especially with leafy vegetable crops, as plant morphology affects plant cutting behaviour. The crop parameters of fenugreek obtained prior to harvesting are shown in Table 5. In the first harvesting stage, average plant height and mean stem diameter were 27.92 cm and 1.89 cm respectively. The row-to-row spacing between the plants and the bed spacing in between were 15 cm and 32 cm, respectively and the stem moisture content was 8.4%. In the 2nd harvesting stage the average plant height was 28.58 cm and stem diameter mean increased 2.21 cm keeping the row spacing at 15 cm and the bed spacing at 30 cm. The stem moisture content after recording was 8.6%. Similarly, at the stage of the third harvesting, the mean plant height was 26.88 cm and mean stem diameter was 2.10 cm with row spacing and bed spacing being 15 and 30 cm, respectively. The moisture content of the stem at this stage was 8.2%. These crop parameters affect cutting resistance and harvesting efficiency as plant height, stem diameter, and moisture content have an effect on interaction among crop stems and cut mechanism. Similar observations have been made in studies on harvesting leafy vegetables, where the crop morphology has a significant influence on the cutting performance and harvesting performance^27–29^.

**Table 5:** Average observation of the crop parameters.

Crop parameters	Particulars
*First time harvesting*	
Avg. plant height, (cm)	27.92
Avg. stem diameter, (mm)	1.89
Avg. row to row spacing, (cm)	15
Avg. distance between beds, (cm)	32
Moisture content in stems, (%)	8.4
*Second time harvesting*	
Avg. plant height, (cm)	28.58
Avg. stem diameter, (mm)	2.21
Avg. row to row spacing, (cm)	15
Avg. distance between beds, (cm)	30
Moisture content in stems, (%)	8.6
*Third time harvesting*	
Avg. plant height, (cm)	26.88
Avg. stem diameter, (mm)	2.1
Avg. row to row spacing, (cm)	15
Avg. distance between beds, (cm)	30
Moisture content in stems, (%)	8.2

**Fig. 4:**
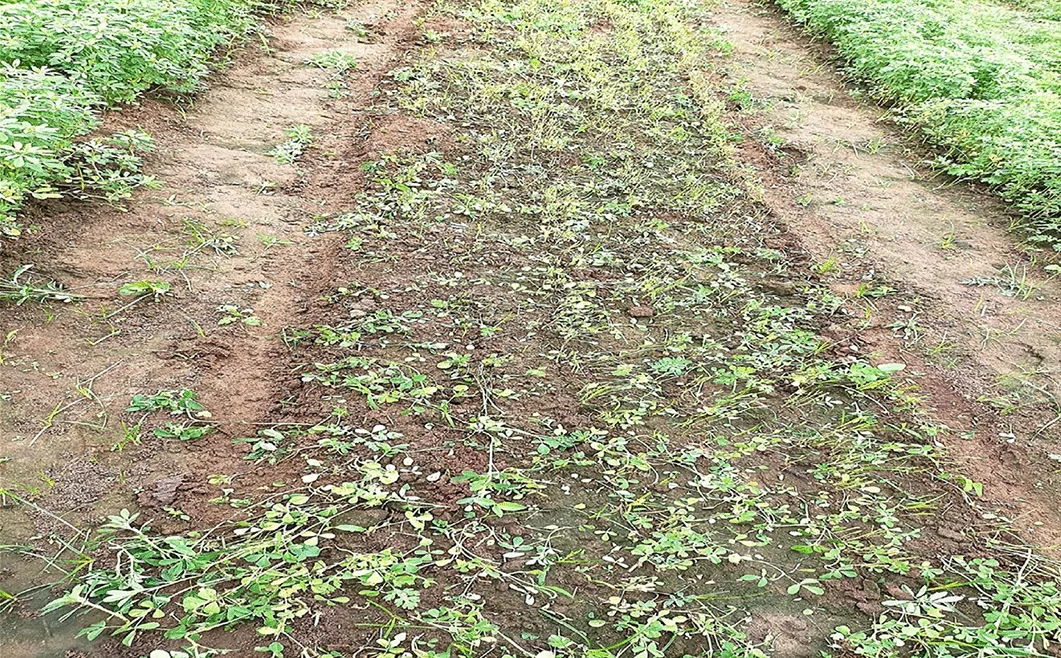
Field condition after harvesting operation showing uniform cutting and residual crop stubble under the cut-and-come-again system.

The developed fenugreek harvester was evaluated under field conditions to assess its harvesting performance. The machine resulted in uniform cutting and effective biomass collection, demonstrating its suitability for cut-and-come-again production systems (Fig. 4).

### Evaluation of machine variables for fenugreek crop

The performance of developed self-propelled battery operated fenugreek harvester was tested under field conditions to determine the effect of few harvesting parameters on the harvesting performance. The evaluation has been done by varying the forward speed, driving pulley of the conveyor, cut height based on the factorial design of experiments adopted for the study. Observations were made of the key performance parameters such as cutting efficiency, conveying loss and harvesting efficiency. The experimental data collected from the field trials were subjected to statistical analysis in an attempt to derive the effect of individual variables as well as their interaction effects on the machine performance. The adequacy and importance of the regression models developed were tested with the analysis of variance (ANOVA). The results of the statistical analysis were then used for the interpretation of the effect of operational parameters on the performance of the developed fenugreek harvester.

#### Model adequacy and ANOVA analysis

The obtained experimental data by the factorial randomized block design were analyzed using Design-Expert software package to assess the effect of forward speed, conveyor driving pulley diameter, and cutting height on the harvesting performance parameters of the developed fenugreek harvester. Analysis of variance (ANOVA) was used to establish the statistical significance of the developed regression models and its adequacy for cutting efficiency, conveying loss, and harvesting efficiency. The results of the analysis of variance (ANOVA) showed that developed regression models were found to be statistically significant for all the performances response considered in the present study. The model F-values found for the various responses showed that the variation explained by the models was significant greater than the unexplained variation. The corresponding p-values were less than 0.05 which indicated that the operational parameters that were considered in the study had significant influence on the harvesting performance of developed machine. The coefficients of determination (R^2^ values) for the developed models showed a high level of agreement between considered experimental and predicted values that reflected reliability of the regression models. The adjusted R^2^ and the predicted R^2^ values were obtained as in good agreement suggesting good predictive capability of the developed models. Furthermore the lack-of-fit tests were found to be non-significant and this indicated that the developed regression models were sufficient to represent the experimental data. The statistical adequacy of the developed models has confirmed that the selected operational parameters had a significant impact on the harvesting performance of the fenugreek harvester. Therefore, the models were deemed suitable to analyse the effect of operational variables and identification of optimal operating conditions to achieve efficient harvesting.

#### Cutting efficiency

Cutting efficiency of the developed fenugreek harvester varied between 97.27 to 98.98%, under all treatment combinations as presented in Table 6 which clearly showed a high and uniform cutting performance with varying values of forward speeds, conveyor driving pulley diameters and cutting heights. The narrow range of variation means stable operation of the cutter mechanism in a wide range of kinematic conditions.

**Table 6:** Average of cutting efficiency (%) at different levels of variables.

S. no	Forward speed, (km/h)	Conveyor driving pulley size, (mm)	Cutting efficiency, (%)	
			Cutting height = 50 mm	Cutting height = 75 mm
1	1.5	101.6	97.27	97.42
2		76.2	97.28	97.48
3		50.8	97.33	97.51
4	2	101.6	97.9	98.06
5		76.2	97.10	98.13
6		50.8	97.96	98.19
7	2.5	101.6	98.59	98.87
8		76.2	98.62	98.89
9		50.8	98.68	98.98

A steady improvement in cutting efficiency was recorded with increase in forward speed from 1.5 to 2.5 km hr^−1^. Maximum value (98.98%) was observed at forward speed of 2.5 km hr^−1^, pulley diameter of 50.8 mm and cutting height of 75 mm (Table 6). This improvement could be explained by an increase in dynamic shearing at greater forward speeds, when the increased relative velocity between the cutter bar and the fenugreek stems results in less deflection of the stem just before severance and enables precise cutting. At lower forward speeds, longer residence time of stems in front of the cutter bar, which fosters partial bending instead of immediate shearing and hence inefficientness in terms of efficiency with slightly higher. Such kinds of kinematic interactions between the machine motion and the crop characteristics have been observed in research dealing with the cutting mechanics^30,31^. The impact on cutting efficiency of conveyor driving pulley diameter was relatively small for the range of values of diameter 50.8—101.6 mm tested (Table 6). Only slight differences in efficiency were found, suggesting that cutting process is rather independent from the dynamics of the conveyor within the chosen operation region. This functional decentralization of the cutting and conveying machine parts guarantees that any changes in conveyor speed would not have a detrimental effect on the cutting performance, thus achieving the stability of the operation during changing operating conditions in the field^31^.

Cutting height had a moderate and consistent effect on cutting efficiency. Increasing the cutting height from 50 to 75 mm produced a visibly better efficiency (Table 6), that can be explained by less rigidity of the stems and low cutting resistance at higher positions along the plant stem. Basal stem regions are found to have a higher structural strength as they are lignified and so require more force to sever than cutting at more elevated positions where there is less resistance and thus allowing easier operation of cutter bars. In addition, enhanced cutting height help reduce soil interference on the cutter assembly thereby minimizing impact loading and contributing to improvement in the cutting uniformity as well as reduction in losses caused by vibration. Similar effects of the cutting height to harvesting performance have been reported for leafy crop systems^31,32^.

**Fig. 5:**
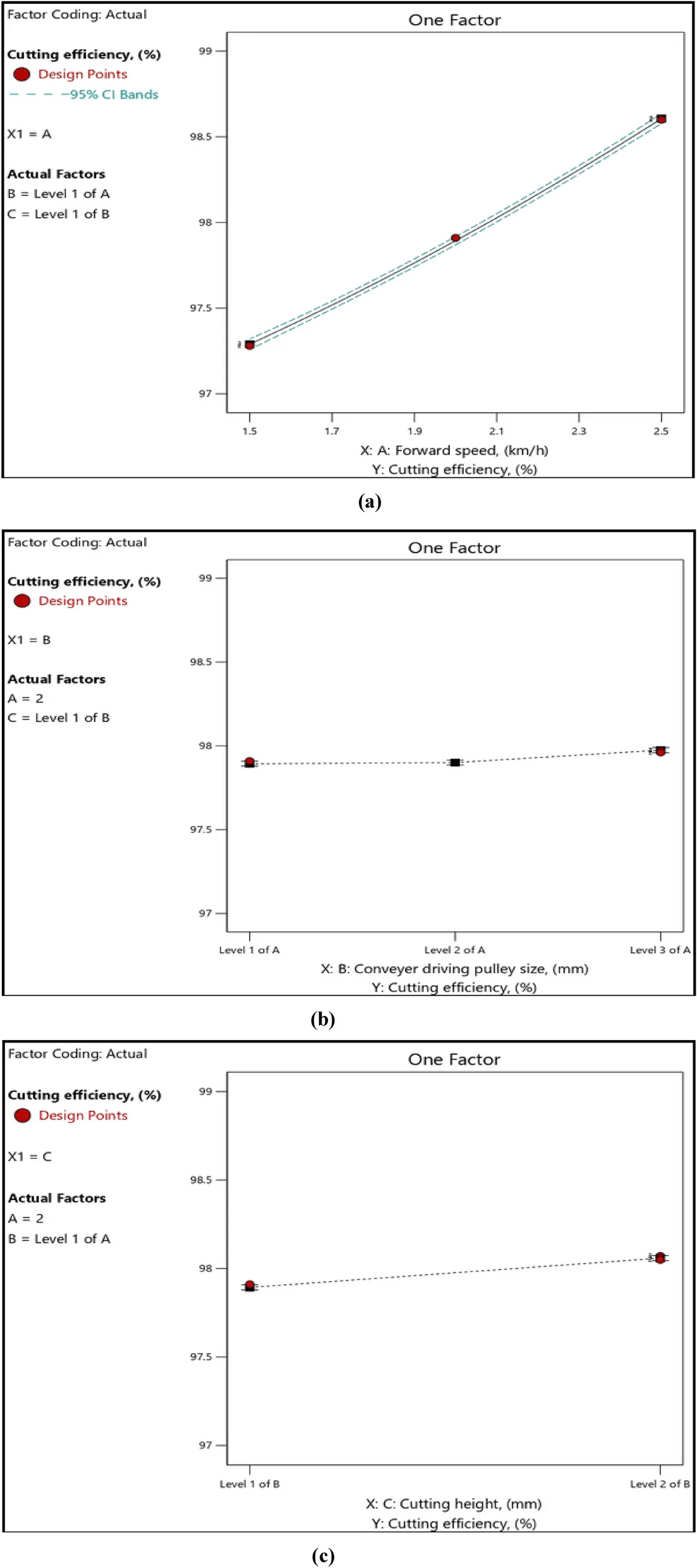
Effect of individual operational parameters on cutting efficiency of the developed fenugreek harvester: (**a**) forward speed (km h^−1^), showing a positive influence due to enhanced dynamic shearing; (**b**) conveyor driving pulley diameter (mm), showing minimal effect within the tested range; and (**c**) cutting height (mm), showing improved efficiency at higher cutting positions due to reduced stem resistance.

Cutting efficiency increased with forward speed (Fig. 5a), which confirms the dominant effects on cutting performance by forward speed. a Effects of operational parameters on cutting efficiency b Cutting efficiency increased with increasing feed rate c Cutting efficiency decreased with increasing cutting depth d Cutting efficiency decreased with increasing number of passes. In contrast, the effects of conveyor driving pulley diameter variation showed small influence on the efficiency of cutting (Fig. 5b), suggesting the weak dependence on conveyor kinematics. Cutting height had a moderate positive effect (Fig. 5c), with higher cutting height being associated with higher efficiency resulting from reduced stem resistance and minimised soil interference.

The statistical analysis provided further confirmation of the significance of the operational parameters on cutting efficiency which is presented in Table 7. The regression model was found to be very significant (*p* < 0.0001) with a response of 0.9995, which shows a magnificent relationship between experimental and predicted values. Among the factors, forward speed (A) had the most dominant influence followed by cutting height (C) and pulley diameter (B). (B) Interaction between forward speed and cutting height (AC) was also found to be significant which indicates the combined effect of forward speed and cutting height on cutting performance. The non-significant lack-of-fit further proves that the model is sufficient for the domain of the experiment.

**Table 7:** Influence of design variables on cutting efficiency of experimental developed harvester (ANOVA) for fenugreek.

Source	Sum of square	DF	Mean squares	F-value	*P*-value	
Model	6.18	10	0.6176	1739.8	< 0.0001	Significant
A-Forward speed, (km/h)	5.47	1	5.47	15,401.1	< 0.0001	
B-Conveyor driving pulley size, (mm)	0.0255	2	0.0127	35.87	< 0.0001	
C-Cutting height, (mm)	0.2236	1	0.2236	629.98	< 0.0001	
AB	0.0025	2	0.0012	3.49	0.0757	
AC	0.0035	1	0.0035	9.94	0.0117	
BC	0.0022	2	0.0011	3.13	0.0931	
A^2^	0.0097	1	0.0097	27.28	0.0005	
Residual	0.0032	9	0.0004			
Lack of fit	0.0026	4	0.0006	4.96	0.0544	Not significant
Pure error	0.0006	5	0.0001			
Cor total	6.18	19				
Std. dev		0.0188	R^2^		0.9995	
Mean		98.05	Adjusted R^2^		0.9989	
C.V. %		0.0192	Predicted R^2^		0.9951	
			Adequate Precision		122.571	

The regression equations relating cutting efficiency under various operating conditions are given in the following Eqs. (1)–(6), showing the quadratic relationship between forward speed and cutting efficiency, where the effect of forward speed is more visible at higher operating values.

For 101.6 mm pulley diameter:

1 $${\mathrm{CE}} = 96.1772 + 0.361425{\mathrm{A}} + 0.244782{\mathrm{A}}^{2}$$

2 $${\mathrm{CE}} = 96.207 + 0.445011{\mathrm{A}} + 0.244782{\mathrm{A}}^{2}$$

For 76.2 mm pulley diameter:

3 $${\mathrm{CE}} = 96.217 + 0.346345{\mathrm{A}} + 0.244782{\mathrm{A}}^{2}$$

4 $${\mathrm{CE}} = 96.2969 + 0.429931{\mathrm{A}} + 0.244782{\mathrm{A}}^{2}$$

For 50.8 mm pulley diameter:

5 $${\mathrm{CE}} = 96.1276 + 0.428782{\mathrm{A}} + 0.244782{\mathrm{A}}^{{2}}$$

6 $${\mathrm{CE}} = 96.172 + 0.512368{\mathrm{A}} + 0.244782{\mathrm{A}}^{2}$$

**Fig. 6:**
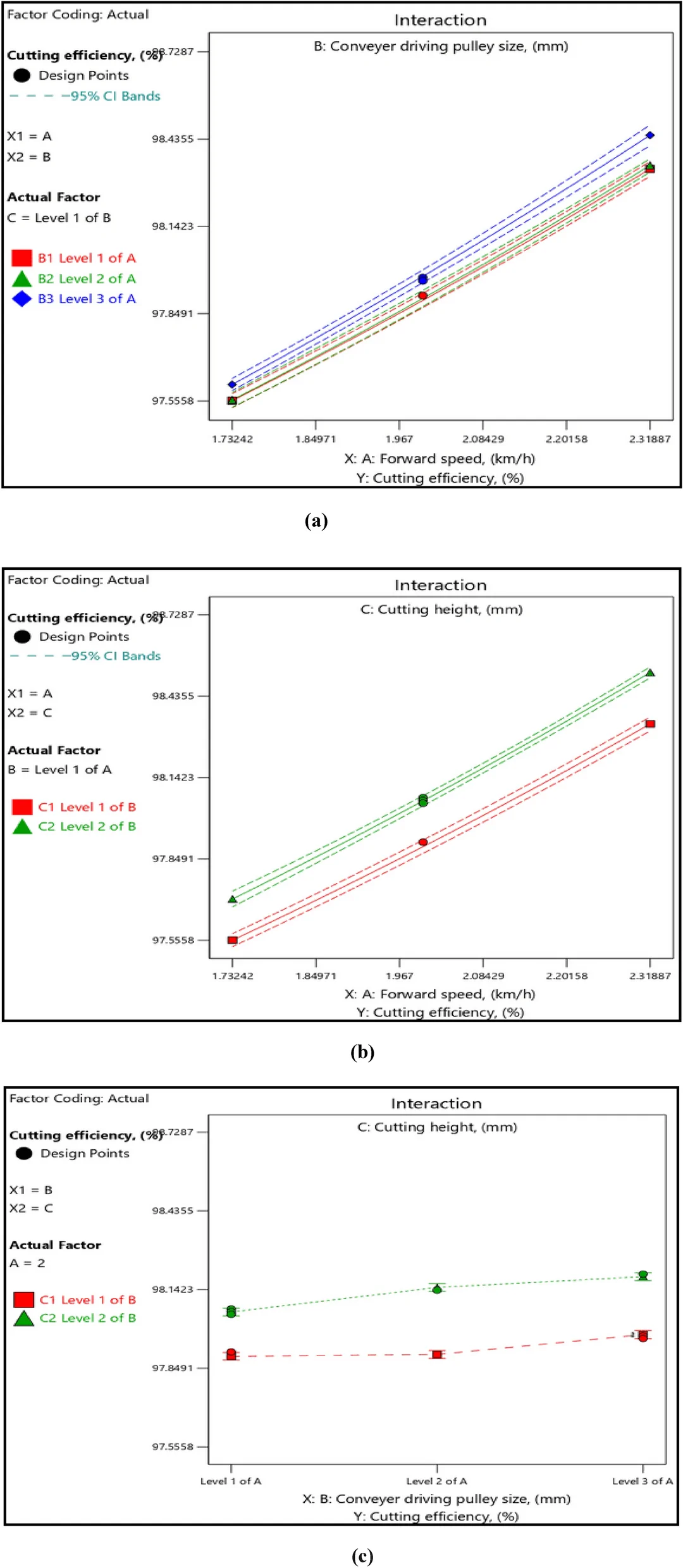
Interaction effects of operational parameters on cutting efficiency: (**a**) forward speed × pulley diameter; (**b**) forward speed × cutting height; and (**c**) pulley diameter × cutting height, indicating combined kinematic influence on cutting performance.

The interaction effects provide further insight into the overall effect of operational parameters interaction. Cutting efficiency was noticeably improved with forward speed for any pulley diameter as was shown in Fig. 6a, which confirms the main role of forward speed regardless of conveyor configuration. Slightly higher efficiencies recorded in small pulley diameters (50.8 mm) may be due to better synchronizing of forward motion and cutter engagement, thereby better crop interception. Similarly, the interaction of forward speed with cutting height as indicated in Fig. 6b suggests that the positive effect of forward speed is increased with cutting height. At 2.5 km h^−1^ and cutting height of 75 mm, the cutting efficiency reached to 98.13% comparing with lower values at reduced cutting heights owing to reduction in stem resistance and improving of cutting conditions. Furthermore the combination effect of cutting height and pulley diameter as shown in Fig. 6c shows the highest cutting efficiency (98.98%) at 50.8 mm pulley diameter and 75 mm cutting height, which indicates optimum coordination between operational parameters.

From a mechanistic perspective, the observed improvements in cutting efficiency may be explained by an increased synchronization between the forward speed, cutter bar reciprocation, and crop flow dynamics. Proper synchronization helps minimize bending of stems before cutting as well as helps in effective shear action and reducing the uncut or partially cut plants. The fenugreek stems viscoelastic behaviour allows favourable response during increased dynamic loading and thus helpful for efficient cutting during optimized operation condition. Similar mechanisms have been reported by work in cutting mechanics as well as work regarding harvesting system kinematics^30,31,33^.

#### Conveying loss (%)

Conveying loss of developed fenugreek harvester showed a variable loss ranging from 0.91% to 1.38% across the combinations of treatments as showed in Table 8, showing minimal loss of harvested biomass during conveying operations. The relatively low magnitude and narrow variation range indicate that the conveyor system operated quite efficiently in various operational conditions, ensuring good material handling.

**Table 8:** Average of conveying loss at different levels of variables.

S. no	Forward speed, (km/h)	Conveyor driving Pulley size, (mm)	Conveying loss, (%)	
			Cutting height = 50 mm	Cutting height = 75 mm
1	1.5	101.6	1.27	1.33
2		76.2	1.08	1.21
3		50.8	0.91	0.98
4	2	101.6	1.31	1.36
5		76.2	1.07	1.23
6		50.8	0.94	1
7	2.5	101.6	1.32	1.38
8		76.2	1.12	1.25
9		50.8	0.98	1.02

An increase in the forward speed from 1.5 to 2.5 km h^−1^ led to a gradual increase in conveying loss (Table 8). The conveying loss has increased from 1.27% to 1.32% for largest pulley diameter (101.6 mm) and followed a similar increasing trend for other pulley sizes. This behaviour can be attributed to the higher momentum of cut biomass at higher forward speeds, reducing the residence time of biomass on the conveyor and so increasing the chances of slippage/spillage before complete transfer. In addition, an increased forward speed may cause a loss of synchronization between the cutting and conveying operations, causing inefficient material handling. Similar trends can be found by harvesting systems in which higher operational speed generated higher conveying or gathering losses due to less material controlling^34,35^.

The change in the diameter of conveyor driving pulleys in terms of covering the conveying loss was found to be extremely significant (Table 8). Conveying loss also increased with an increase in pulley diameter from 50.8 mm to 101.6 mm and losses were observed to be minimum at the smallest pulley diameter. For example, at forward speed 1.5 km h^−1^ and cutting height 50 mm, conveying loss was 0.91% for 50.8 mm pulley as compared to 1.27% for 101.6 mm pulley. This trend can be explained by greater conveyor belt velocity with greater pulley diameter that causes instability in biomass transport and greater slippage of material. At higher conveyor speeds the cut crop tends to lose contact with the conveyor surface due to lack of sufficient frictional resistance and hence will have higher losses. Similar results have been observed in harvesting systems based on conveyor technology, where high belt speed has a negative effect on the retention of the material^34^.

Cutting height also had a reasonable effect on conveying loss. Increasing the processing height from 50 to 75 mm led to a small rise in the conveying loss (Table 8). This may be attributed to the fact that elevation at which crop material enters the conveyor system is increased which leads to higher drop height and higher impact forces on transferring crop material. Additionally, increased cutting height may change the path of should biomass flow, decreasing capture efficiency of the crop by the conveyor system. Comparable observations have been reported in the studies where cutting height has been studied as influencing the flow dynamics and conveying efficiency of the crop^35^.

**Fig. 7:**
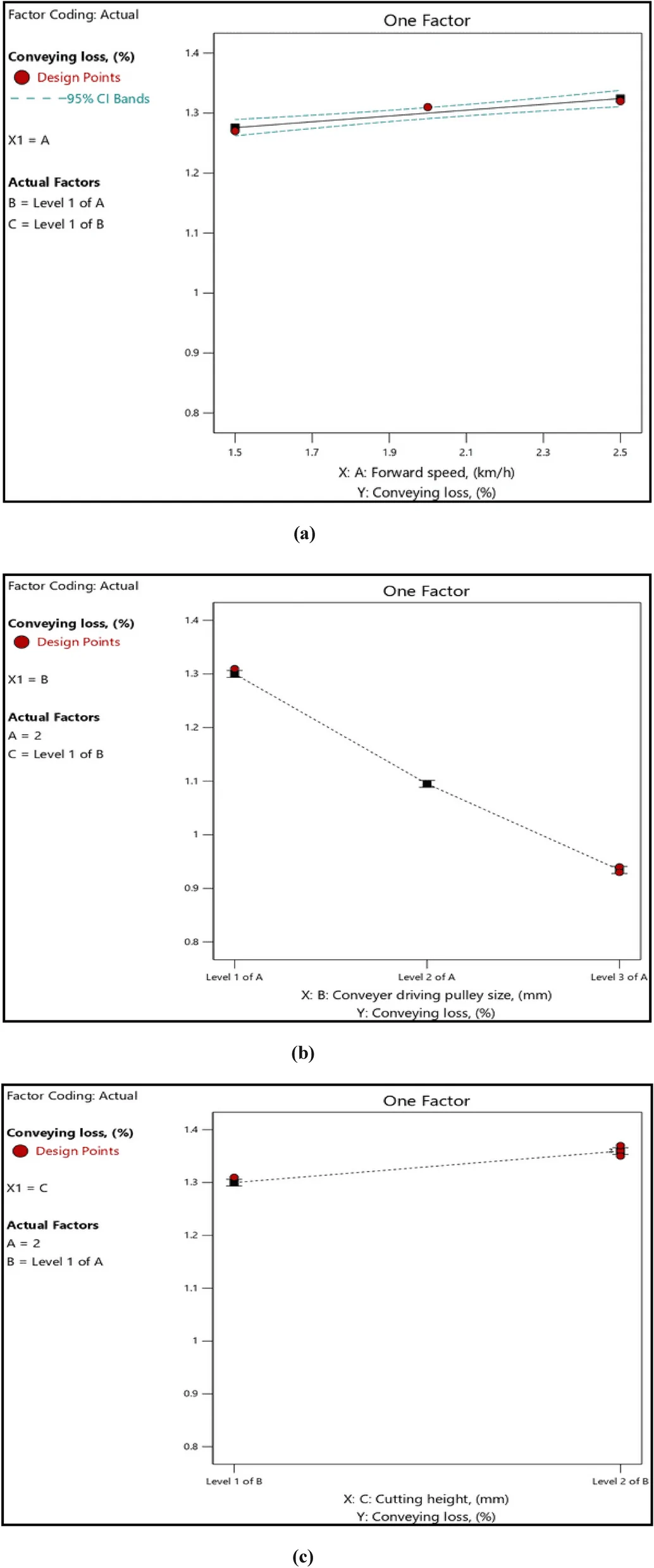
Effect of individual operational parameters on conveying loss: (**a**) forward speed (km h^−1^), showing increasing loss with speed; (**b**) conveyor driving pulley diameter (mm), showing increased loss with larger diameters; and (**c**) cutting height (mm), showing a slight increase in loss at higher cutting heights.

The individual effects of the operational parameters on conveying loss are shown in (Fig. 7 a–c). Forward speed increased the conveying loss (Fig. 7a), which confirms the effect on the efficiency of material handling. A significant increase in the conveying loss was found for increasing pulley diameter (Fig. 7b) suggesting a strong dependence on the conveyor kinematics. Cutting height had a moderate positive influence on conveying loss (Fig. 7c), in which higher cutting height increased losses slightly as a result of biomass entry conditions.

The statistical analysis further proved the importance of operational parameters on the conveying loss (as shown in Table 9). The regression model was determined to be highly significant (*p* < 0.0001) with a coefficient of determination (R^2^ = 0.9995) indicating that the match between experimental and predicted values was very good. Among the factors, pulley diameter (B) had the dominance most, followed by cutting height (C) and forward speed (A). The interaction term (BC) was also found to be significant indicating that the effect of pulley diameter and cutting height combines to have a critical role in determining conveying loss. The further proof of adequate of the developed model in the experimental range is the lack of significant lack-of-fit.

**Table 9:** Influence of design variables on conveying a loss of experimental developed harvester (ANOVA).

Source	Sum of squares	DF	Mean square	F-value	*P*-value	
Model	0.5126	10	0.0513	1128.51	< 0.0001	Significant
A-Forward speed, (km/h)	0.0048	1	0.0048	106.71	< 0.0001	
B-Conveyor driving pulley size, (mm)	0.4170	2	0.2085	4589.72	< 0.0001	
C-Cutting height, (mm)	0.0350	1	0.0350	771.32	< 0.0001	
AB	0.0000	2	5.096E-06	0.1122	0.8951	
AC	0.0000	1	0.0000	0.4324	0.5273	
BC	0.0041	2	0.0020	44.96	< 0.0001	
A^2^	0.0001	1	0.0001	2.85	0.1255	
Residual	0.0004	9	0.0000			
Lack of fit	0.0002	4	0.0000	0.7620	0.5923	Not significant
Pure error	0.0003	5	0.0001			
Cor total	0.5130	19				
Std. dev		0.018	R^2^		0.9995	
Mean		98.05	Adjusted R^2^		0.9989	
C.V. %		0.019	Predicted R^2^		0.9951	
			Adequate Precision		122.5714	

The regression equations for conveying loss under various operating conditions are listed in the following Eqs. (7)–(12), indicating a quadratic dependence between the forward speed and conveying loss and, thus, the nonlinear interaction between operational parameters and the material flow behaviour.

For 101.6 mm pulley diameter:

7 $${\mathrm{CL}} = 1.09997 + 0.159005{\mathrm{A}} - 0.0283153{\mathrm{A}}^{2}$$

8 $${\mathrm{CL}} = 1.17931 + 0.152768{\mathrm{A}} - 0.0283153{\mathrm{A}}^{{2}}$$

For 76.2 mm pulley diameter:

9 $${\mathrm{CL}} = 0.90966 + 0.15534{\mathrm{A}} - 0.0283153{\mathrm{A}}^{2}$$

10 $${\mathrm{CL}} = 1.04977 + 0.149103{\mathrm{A}} - 0.0283153{\mathrm{A}}^{2}$$

For 50.8 mm pulley diameter:

11 $${\mathrm{CL}} = 0.734512 + 0.159399{\mathrm{A}} - 0.0283153{\mathrm{A}}^{2}$$

12 $${\mathrm{CL}} = 0.811655 + 0.153163{\mathrm{A}} - 0.0283153{\mathrm{A}}^{2}$$

**Fig. 8:**
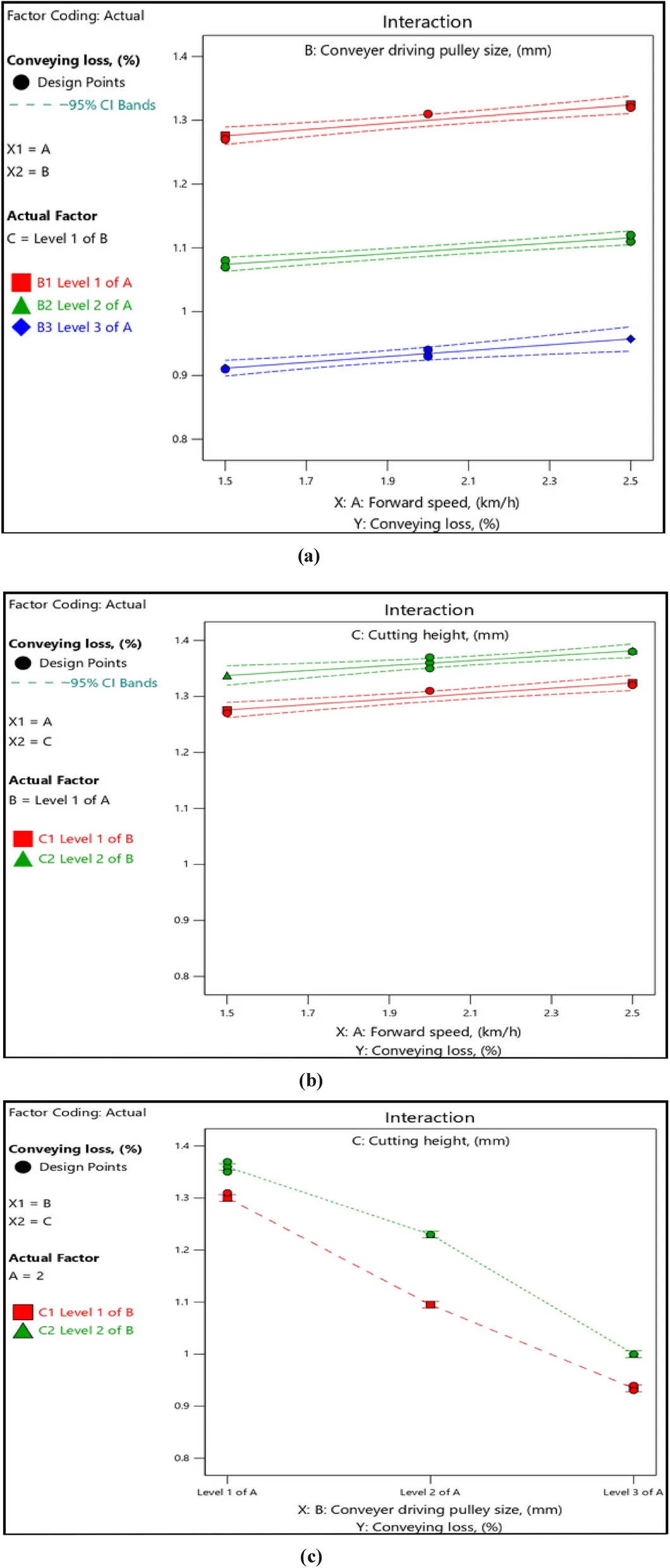
Interaction effects of operational parameters on conveying loss: (**a**) forward speed × pulley diameter; (**b**) forward speed × cutting height; and (**c**) pulley diameter × cutting height, indicating combined influence on biomass transfer losses.

These equations show conveying loss is initially increased by forward speed, and depends on normally both conveyor speed and material flow behaviour. The interaction effects add more details on the effect of operational parameters on each other. Conveying loss increased as forward speed and pulley diameter increased as shown in Fig. 8a; showing that a higher conveyor speed when used in conjunction with greater forward velocity results in greater material instability and loss. Similarly, in Fig. 8b, increasing cutting height and forward speed cause a further increase of conveying loss caused by a combination increasing biomass velocity and changing crop entry dynamics.

Furthermore the interaction between the pulley diameter and both the cutting height (Fig. 8c) it can be obtained that the greatest conveying losses were recorded at larger pulley diameters and higher cutting heights. This combination results in a higher conveyor velocity as well as a higher height of biomass drop, leading to a decrease in the control of the material flow and to higher losses.

From a mechanistic point of view, the conveyance of loss is determined by the balance between the conveyor velocity, forward speed and biomass flow dynamics. Efficient conveying requires a coordination of these parameters in order to ensure stable contact between crop material and conveyor surface. Excessive speed of conveyor, i.e. forward speed, disturbs this balance and results in slippage and spillage. The interaction between the dynamics of mechanical transport and crop physical properties is very important in determining conveying efficiency. Similar mechanisms have been reported in harvesting and conveying systems by improper synchronization which lead to increased material losses^34,35^.

#### Harvesting efficiency (%)

Harvesting efficiency of the developed harvester was observed with range of 96.00% to 97.96% for all the treatment combinations as shown in Table 10 indicating effective integration of the cutting and conveying operation under various field conditions. The comparatively small range of the harvesting efficiency is related to the fact that the system is fairly stable and can always perform on the same level, when changing the combination of operation parameters.

**Table 10:** Average of harvesting efficiency at different levels of variables.

S. no	Forward speed, (km/h)	Conveyor driving pulley size, (mm)	Harvesting efficiency, (%)	
			Cutting height = 50 mm	Cutting height = 75 mm
1	1.5	101.6	96	96.09
2		76.2	96.2	96.3
3		50.8	96.42	96.5
4	2	101.6	96.59	96.7
5		76.2	96.83	96.9
6		50.8	97.02	97.19
7	2.5	101.6	97.27	97.49
8		76.2	97.5	97.64
9		50.8	97.7	97.96

A progressive increase in harvesting efficiency was recorded with an increase in forward speed from 1.5 to 2.5 km h^−1^ (Table 10). The maximum harvesting efficiency value, 97.96%, was obtained at a forward speed of 2.5 km h^−1^, at the same time pulley diameter and cutting height obtained from this test were 50.8 mm and 75 mm respectively. This improvement can be attributed to better synchronization between cutting and conveying processes at higher forward speeds which provides for better crop collection with less field losses. At lower forward speeds, poor cutting and slow transfer of biomass to the conveyor could lead to small losses of accumulation and thus reduction in the overall harvesting efficiency. These findings suggest that the harvesting efficiency is highly affected by the coordination of all components of the machine rather than the individual parameters of a single component as well^31^.

The relationship of the conveyor driving pulley diameter to the efficiency of harvesting followed a downward trend after increasing the pulley size (Table 10). In particular, the harvesting efficiency decreased with an increase in the pulley diameter from 50.8 mm to 101.6 mm. For example, at a forward speed of 2.5 km h^−1^ and cutting height of 75 mm, harvesting efficiency decreased from 97.96% at 50.8 mm pulley diameter to 97.49% at 101.6 mm. This decrease is mainly related to greater conveying losses for higher pulley diameters, which will decrease the effective recovery of the harvested biomass. As a cumulative measure of cutting and conveying performance, harvesting efficiency determines the total system output and thus any inefficiency in transporting material is therefore also reflected in the overall system output.

Cutting height showed a positive association with harvesting efficiency, with findings of higher efficiency values of harvesting at 75 mm when compared to 50 mm (Table 10). This improvement is related to increased cutting performance performance at higher positions, with a reduction in stem resistance leading to cleaner cutting and a reduction in uncut plants. In addition, better crop flow from the cutter bar to the conveyor at high cutting heights helps to ensure efficient biomass transfer, which improves the efficiency of the harvesting. Similar interactions between cutting condition and harvesting performance have been found in crop harvesting systems^30,33^.

**Fig. 9:**
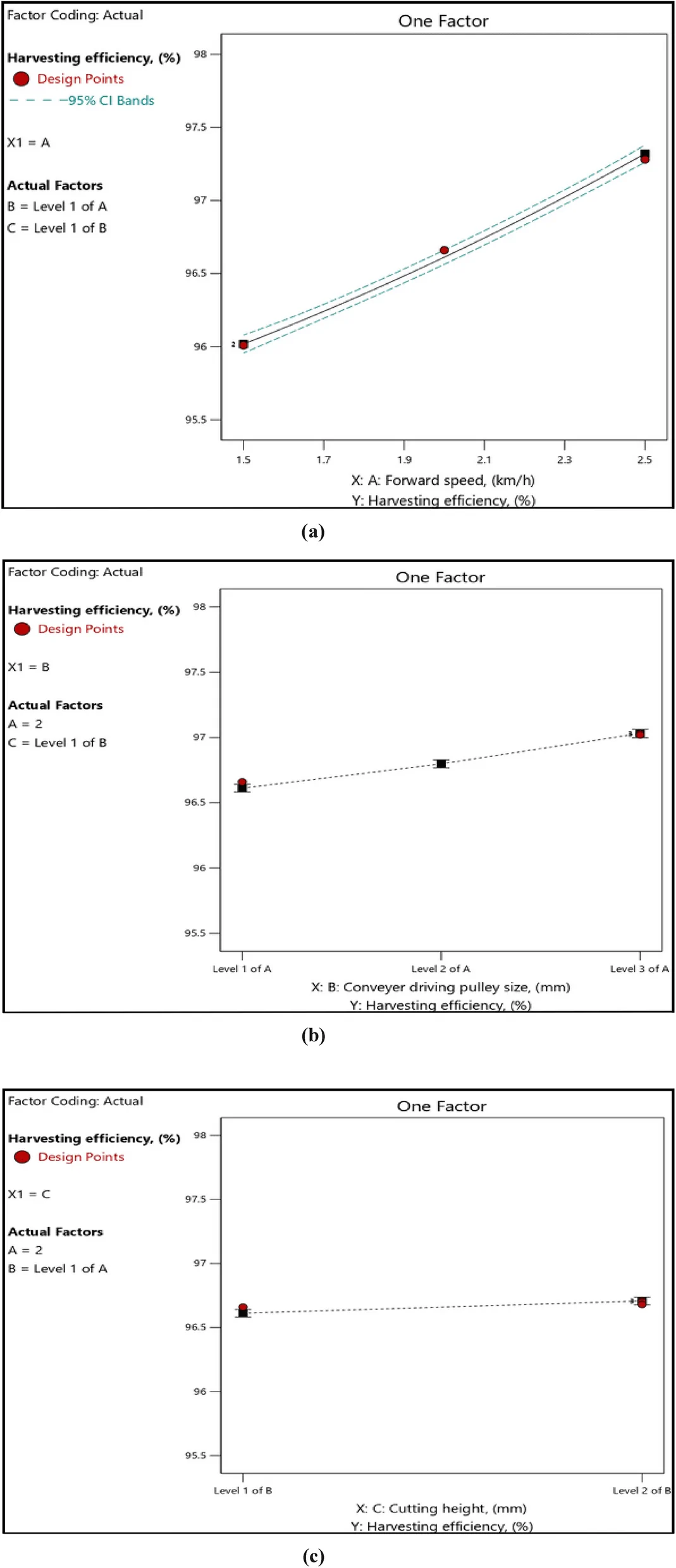
Effect of individual operational parameters on harvesting efficiency: (**a**) forward speed (km h^−1^), showing a positive influence; (**b**) conveyor driving pulley diameter (mm), showing a decreasing trend with larger diameters; and (**c**) cutting height (mm), showing improved efficiency at higher cutting heights.

The influence of the different individual parameters on the harvesting efficiency can be seen in Fig. 9a–c. A clear positive trend with the forward speed is found in Fig. 9a, pointing to a major role in the enhancement of the harvesting performance. In comparison, a slow reduction in harvesting efficiency with increasing pulley diameter is seen in Fig. 9b through the influence of conveying losses. Figure 9c shows that a positive contribution of harvesting efficiency occurs with increasing cutting height because of better cutting and handling conditions of materials.

Statistical analysis of harvesting efficiency presented in Table 11 showed the significance of selected operational parameters. The regression model was found to be very significant (*p* < 0.0001) with a coefficient of determination (R^2^ = 0.9980), which gives good agreement between the observed and predicted values. Forward speed (A) was identified as the most influential factor followed by pulley diameter (B) and cutting height (C). The presence of the quadratic term (A^2^) suggests a non-linear dependence of the relationship between the forward speed and the harvesting efficiency. The non-significant lack-of-fit provides further validation for the suitability of the developed model for predictions of the harvesting efficiency within the experimental range.

**Table 11:** Influence of design variables on harvesting efficiency of experimental developed harvester (ANOVA).

Source	Sum of squares	DF	Mean square	F-value	*P*-value	
Model	5.78	10	0.5780	453.50	< 0.0001	Significant
A-Forward speed, (km/h)	5.18	1	5.18	4061.75	< 0.0001	*
B-Conveyor driving pulley size, (mm)	0.6833	2	0.3416	268.06	< 0.0001	*
C-Cutting height, (mm)	0.0731	1	0.0731	57.38	< 0.0001	*
AB	0.0022	2	0.0011	0.8572	0.4563	*
AC	0.0051	1	0.0051	3.99	0.0769	*
BC	0.0009	2	0.0004	0.3439	0.7179	*
A^2^	0.0133	1	0.0133	10.46	0.0103	*
Residual	0.0115	9	0.0013			
Lack of fit	0.0048	4	0.0012	0.9006	0.5272	Not significant
Pure error	0.0067	5	0.0013			
Cor total	5.79	19				
Std. Dev		0.0357	R^2^		0.9980	
Mean		96.89	Adjusted R^2^		0.9958	
C.V. %		0.0368	Predicted R^2^		0.9888	
			Adequate precision		74.7021	

The regression equations for harvesting efficiency under various operating conditions are given in Eqs. (13)–(18). These equations highlight the existence of a quadratic trend between the forward speed and harvesting efficiency as a result of the nonlinear role of the operational parameters upon the overall system performance.

At 50 mm cutting height:

13 $${\mathrm{HE}} = {95}.{1}0{82} + 0.{1577}0{\mathrm{1A}} + 0.{\mathrm{287126A}}^{{2}}$$

At 75 mm cutting height:

14 $${\mathrm{HE}} = {95}.0{141} + 0.{258}0{\mathrm{46A}} + 0.{\mathrm{287126A}}^{{2}}$$

For 76.2 mm pulley diameter:

At 50 mm cutting height:

15 $${\mathrm{HE}} = {95}.{367} + 0.{\mathrm{131379A}} + 0.{\mathrm{287126A}}^{2}$$

At 75 mm cutting height:

16 $${\mathrm{HE}} = {95}.{2869} + 0.{\mathrm{231724A}} + 0.{\mathrm{287126A}}^{2}$$

For 50.8 mm pulley diameter:

At 50 mm cutting height:

17 $${\mathrm{HE}} = {95}.{4593} + 0.{2}0{\mathrm{7126A}} + 0.{\mathrm{287126A}}^{2}$$

At 75 mm cutting height:

18 $${\mathrm{HE}} = {95}.{4}0{54} + 0.{3}0{\mathrm{7471A}} + 0.{\mathrm{287126A}}^{{2}}$$

**Fig. 10:**
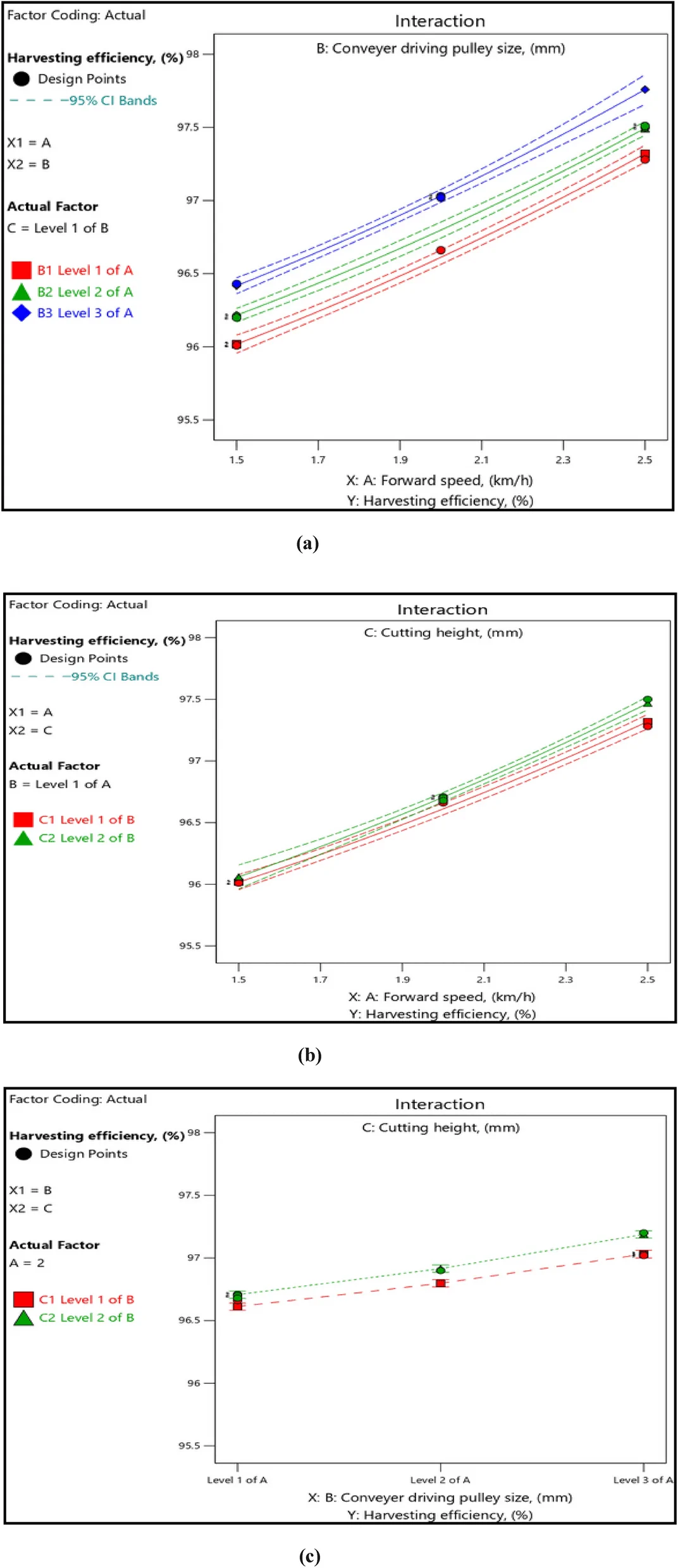
Interaction effects of operational parameters on harvesting efficiency: (**a**) forward speed × pulley diameter; (**b**) forward speed × cutting height; and (**c**) pulley diameter × cutting height, indicating combined influence on overall harvesting performance.

These equations show the efficiency of harvesting to be a function of forward speed in a nonlinear manner, with the quadratic term indicating that as the forward speed is increased the harvesting efficiency will increase at an accelerating rate due to an increasing degree of coordination between operation of the cutting and conveying components of the harvesting machine. The interaction effects are one more clarification of combined behaviour of operational parameters. As can be seen in Fig. 10a, the harvesting efficiency was higher at higher forward speeds coupled with lower pulley diameters implying that optimization is attained at higher forward speeds coupled with lower conveyor velocities. Similarly, Fig. 10b shows that the cutting height has a positive effect on the forward speed resulting in improved harvesting efficiency because of reduction of cutting resistance and improved crop flow dynamics. Furthermore, the effect of pulley diameter and cutting height on the boosting of harvesting efficiency as shown in Fig. 10c shows that the maximum harvesting efficiency was obtained at a smaller pulley diameter and a higher cutting height. This combination allows one to minimize the conveying losses and maximize the cutting effectiveness for maximum overall harvesting performance.

From a mechanistic perspective, harvesting efficiency is the summation of both, cutting efficiency and conveying efficiency, and is therefore determined by the extent of synchronization between these subsystems. Efficient harvesting is achieved when forward speed, cutter bar, and conveyor movement are in optimum coordination to insure total cutting and effective transfer of material. Any imbalance such as high conveyor speed or poor cutting conditions results in increased losses and lower harvesting efficiency. The interplay of machine kinematics and crop characteristics thus plays an important and decisive role in the determination of the overall system performance^30,31^.

#### Optimum value of each independent variable for fenugreek crop

The optimization of the operational parameters was conducted using the desirability function approach in Design Expert software to achieve simultaneous maximum cutting efficiency, as well as harvesting efficiency and minimum conveying loss. The range of responses obtained during experimentation were as cutting efficiency ranging from 97.27% to 98.98%, conveying loss ranging from 0.91% to 1.38% and harvesting efficiency ranging from 96.00% to 97.96% which were summarized in Table 12. These ranges mean that while all the combinations of parameters led to the high performance, there are distinctive differences depending on the interplay between variables.

**Table 12:** Five desirable solutions predicted by Design Expert Optimization for effective harvesting efficiency of the fenugreek harvester.

Forward speed, (km/h)	Conveyor driving pulley size, (mm)	Cutting height, (mm)	Cutting efficiency, (%)	Conveying loss, (%)	Harvesting efficiency, (%)	Desirability	
**1.607**	**101.6**	**75**	**97.628**	**0.985**	**96.64**	**1.000**	**Selected**
2.46	**101.6**	**75**	98.92	1.011	97.91	1.000	
1.500	76.2	75	97.493	1.210	96.28	1.000	
2.000	50.8	75	98.076	1.372	96.67	1.000	
1.500	101.6	75	97.491	0.978	96.51	1.000	
1.500	76.2	50	97.287	1.079	96.21	1.000	
2.000	101.75	75	98.176	1.005	97.16	1.000	

A detailed study of Table 12 reveals that several combinations of parameters provided a desirability value of 1.000, thus equally optimal solutions can be found given the specified constraints. However, among these, the combination of forward speed 2.46 km h^−1^, diameter of pulley 50.8 mm and cutting height 75 mm gave one of the highest performance outputs with cutting efficiency of 98.92%, conveying loss of 1.01% and harvesting efficiency of 97.91%. Compared to lower forward speeds (e.g. 1.5 km/h) where the efficiency of harvesting was around 96.2—96.6%, this represents an improvement of around 1.3–1.7 percentage points which is significant given the already high baseline efficiency of harvesting.

The superiority of this parameter combination can be explained by means of coordinated kinematic behaviour. Increasing forward speed from 1.5 to 2.5 km h^−1^ improves dynamic interaction between cutter bar and crop resulting in improved cutting efficiency as was also seen earlier in Table 6. However, this improvement is only useful when supported by suitable conveyor velocity. At 50.8 mm pulley diameter, the conveyor speed is moderate and therefore contact time between biomass and conveying surface is adequate for several reasons, in turn minimizing conveying loss to 1.01%, which is significantly lesser than values > 1.30% at larger pulley diameters. The role of cutting height is also equally critical. At 75 mm harvesting efficiency is consistently higher than values recorded at 50 mm under most combinations (Table 12) indicating that the cutting conditions were improved and resistance reduced. This is also consistent with previous results showing increased cutting efficiency when cutting was performed at a higher cut position because of a reduced stem flexibility at the higher cut position^35,36^. Consequently, the optimized condition is an amortized balance between reducing conveying loss and reducing conveying efficiency.

**Fig. 11:**
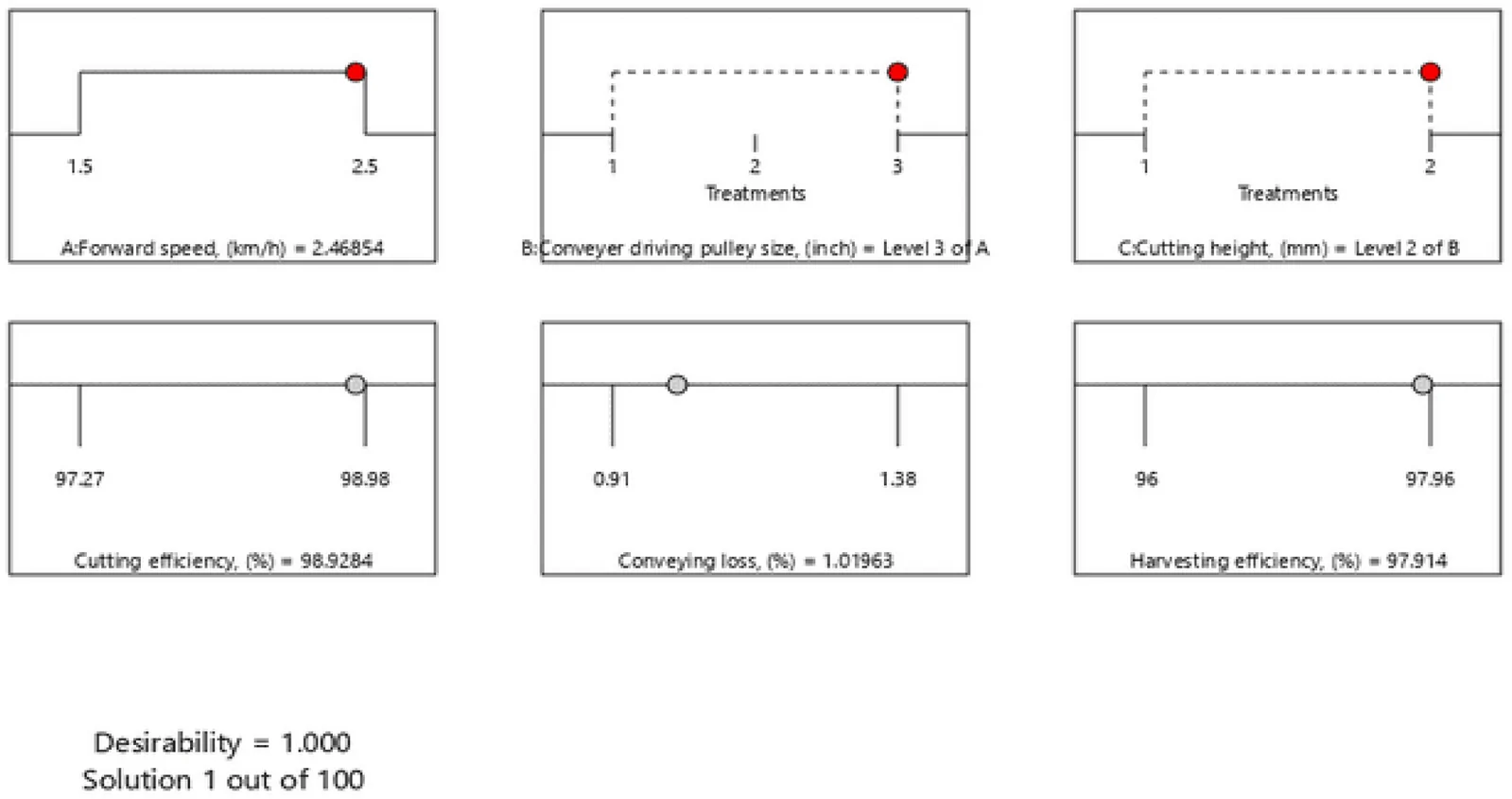
Response surface showing the combined effect of forward speed, pulley diameter, and cutting height on harvesting performance, indicating the optimal operational region for maximum efficiency and minimum loss.

The response surface behaviour of Fig. 11 also supports these findings, showing a clear optimal region with high forward speed, low pulley diameter and high cutting height combining to give a maximum harvesting efficiency. The curved nature of the response surface confirms the nonlinearity of the interaction among the variables in conformity to the quadratic regression models developed earlier.

From the mechanistic point of view, optimized performance is the result of a synchronization between forward motion (V_f_), conveyor velocity (V_c_) and cutter bar dynamics that ensures the continuous flows of crop, without accumulation or spillage. Similar optimization tendencies with harvesting systems have been reported by^31^, in which balanced kinematic coordination was reported to be the key factor to regulate harvesting efficiency, and^30^, which highlighted the importance of dynamic cutting conditions to enhance severance efficiency.

#### Effect of operational parameters on field efficiency of the harvester for fenugreek

**Fig. 12:**
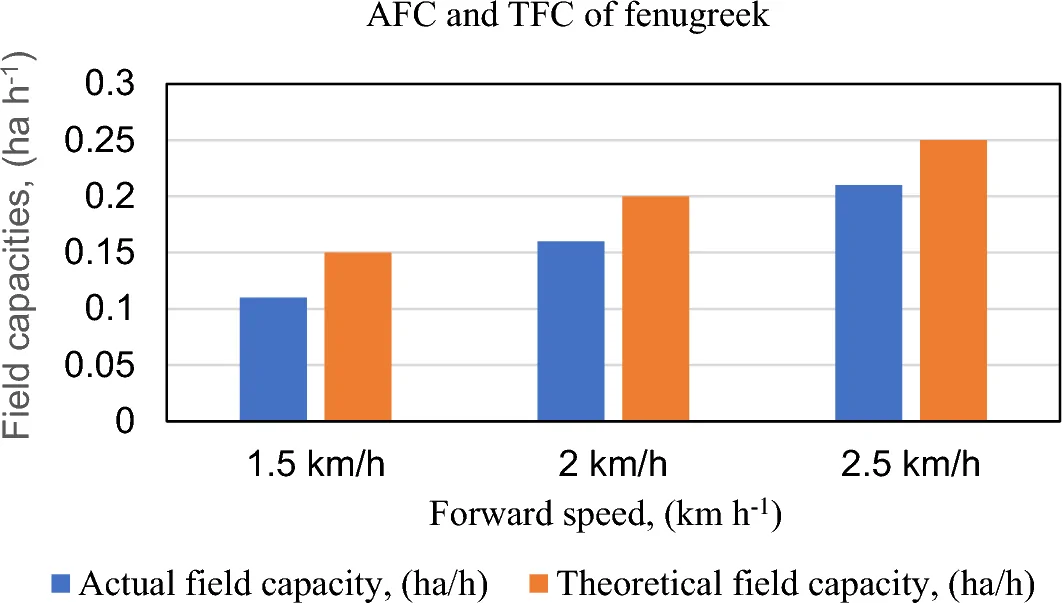
Effect of forward speed on theoretical and effective field capacity of the fenugreek harvester, showing an increasing trend with speed.

**Fig. 13:**
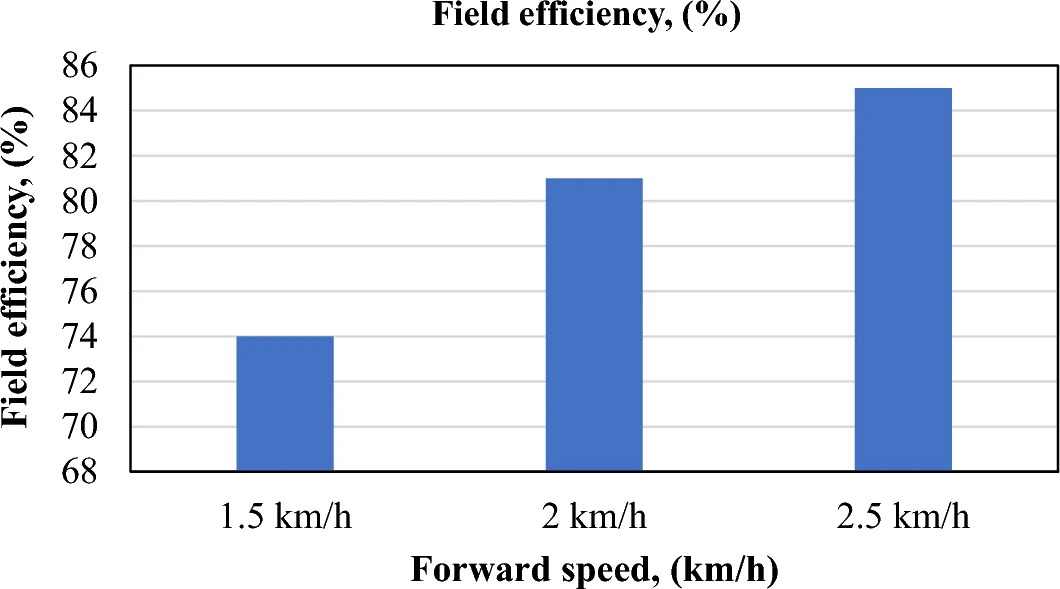
Effect of forward speed on field efficiency of the fenugreek harvester, reflecting variation with changes in operational conditions.

The effect of forward speed on field performance parameters of theoretical field capacity, effective field capacity and field efficiency are illustrated in Figs. 12 and 13 . A definite and consistent rise in both theoretical and effective field capacity was noticed with an increase in forwards speed. As shown in Fig. 12 the effective field capacity rose from 0.11 ha h^−1^ at 1.5 km h^−1^ to 0.21 ha h^−1^ with 2.5 km h^−1^ and is approx. 91% more for field capacity. This increase is in direct proportion to forward speed as the theoretical field capacity is a relationship to the speed of travel and the cutting width. The developed harvester had a higher field capacity than the rotary leafy vegetables harvester reported by^26^ mainly because of the larger effective cutting width and the so far improved material handling efficiency. In addition to field capacity, field efficiency also increased as the forward speed increased, as shown in Fig. 13. Field efficiency increased from 74% at 1.5 km h^−1^ to 85% at 2.5 km h^−1^, and therefore improved utilization of operational time. This increase may be explained by the decrease in relative nonproductive time (turning, adjustments and minor delays) with increasing forward speed. At lower speeds, these losses in time are a higher proportion of the total operational time and systems will therefore be less efficient. The trend shown in Fig. 13 has shown that the rate of increase in field efficiency is not linear, but that the field efficiency flattens out at higher speeds, suggesting that at a certain point, that increases in speed may not have a significant effect on the efficiency. This behaviour is in line with characteristics of field machinery performance where operational efficiency depends on the balance between productive and non-productive components of time. The developed harvester showed better efficiency in the field, compared to the tractor PTO-operated leafy vegetable harvester reported by^37^, which operated at a higher speed but gave less efficiency due to higher loss of power in turning and complexity of the machine. The tight and self-propelled structure of the present harvester makes it possible to be more maneuverable, which can be seen in the reduction of the turning radius, but also in its adaptation to the small plot conditions typical of the fenugreek cultivation. From a mechanistic point of view, the resulting improvement in field efficiency with forward speed is related to the improvement in operational continuity with less idle time. However, it is important to note that excessively high forward speed beyond the optimum range may negatively impact cutting and conveying performance causing increased losses. Therefore, although the governing speed of higher speeds is beneficial to improve the field capacity and efficiency, it must be properly optimized to ensure the overall harvesting performance. These results concur with those of^28,34,35,38^ which found that the forward speed has a significant effect on the field capacity and operational efficiency, but have to be balanced with other machine performance parameters to obtain optimal results.

#### Performance of the harvester at the optimized machine variable of fenugreek crop

The experimental performance of the developed self-propelled battery operated fenugreek harvester under the optimized operational conditions found in Section "Optimum value of each independent variable for fenugreek crop" has been tested and the results are reported in Table 13. Under these conditions, cutting efficiency of the machine was 98.1%, harvesting efficiency was 97.15% and conveying loss is 0.95%, which means efficient harvesting with minimum loss. Comparing the fixed values of the mentioned range of values for all treatment combinations (Tables 3, 4, 5, 6 and 7), the optimized condition was found to end with higher efficiencies and lower losses, validating the success of the desirability-based parameter optimization. This improvement corresponds with improvement in the synchronicity of cutting and conveying operations resulting in stable flow of crops and reduced material loss. The theoretical field capacity was 0.16 ha h^−1^ and the effective field capacity was 0.13 ha h^−1^, which is the field efficiency of 81.25%. The reason that the theoretical and effective capacities are relatively close is indicative of efficient utilization of machines with limited operational losses during actual field needs. The harvester worked for a continuous 6.5 h on a single battery charge, proving its suitability to work in practical field conditions and as a way of reducing dependency on manual labour and conventional fuel-based systems. From a mechanistic point of view the higher performance under optimum conditions can be explained by good synchronization between advance velocity, cutter bar action and conveyor velocity. This synchronization reduces the bending of the stems while cutting and assures an efficient transfer of biomass, reducing losses and increasing the efficiency of the harvesting activity. Similar enhancements of performance based on parameter optimization have been reported in harvesting systems^30,31,35^.

**Table 13:** Performance of the harvester at the optimized machine variable of fenugreek crop.

S. no.	Parameters	Average
1	Harvesting efficiency, (%)	97.15
2	Cutting efficiency, (%)	98.1
3	Conveying loss, (%)	0.95
4	Theoretical field capacity, (ha/h)	0.16
5	Actual field capacity, (ha/h)	0.13
6	Field efficiency, (%)	81.25
6	Working hours, (h)	6.5

### Effect of harvesting with developed harvester on yield of selected crops

The yield of fenugreek was 30.7 q/ha in the first-time harvesting using the developed self-propelled battery-operated fenugreek harvester after 31 days after sowing (DAS). Subsequent second time yield was 24.2 q ha^−1^ at 47 days after sowing (DAS) and third time yield was found to be 20.3 q ha^−1^ at 61 DAS. These results when compared with the yield of^39^ show significant change being found. The higher yield was attributed to the utilization of broadcast method, high seed rate, use of hybrid seed and timely supply of water and plant requirements.

### Economic evaluation of the developed self-propelled battery-operated fenugreek harvester

The economic performance of the developed battery-operated fenugreek harvester was studied using the standard cost analysis procedures and the assumptions involved in estimating the fixed and operating costs are given in Table 4. The developed harvester brought down the harvesting cost to the tune of 7,202.33 ₹ ha^−1^ which corresponds to a reduction of 96.03% compared to manual harvesting thereby bringing down a substantial economic gain. Labour requirement was significantly reduced by 194.57 labour hours per hectare, which resulted in a saving of 97.28% of harvesting time which is of great importance under conditions of labour deficiency and time-sensitive harvesting operations. The break-even was calculated at 450.89 h per annum, which represents economic viability in the moderate annual use while the payback time was calculated at 2.94 years, which represents quick recovery of the initial investment. The benefit and cost ratio was calculated to be 39.04 which shows highly profitable operation. The improved economic performance is directly associated to machine technical efficiency under optimized condition, high cutting efficiency (98.1%), harvesting efficiency (97.15%) and low conveying loss (0.95%) were achieved, with maximum output and minimum losses. Similar enhancements in economic viability by mechanized harvesting and optimization of parameters have been reported in other studies^30,33,35^.

## Conclusion

A self-propelled battery-operated fenugreek harvester was designed and tested under field condition for cut and come again production systems. Consistently high performance was achieved by the machine with cutting efficiency (97.27–98.98%), conveying loss (0.91–1.38%) and harvesting efficiency (96.00–97.96%) were recorded during all the operational combinations. Operational parameters had a significant effect (p < 0.05) with forward speed having the greatest influence on cutting and harvesting efficiency and pulley diameter determining conveying loss. The regression models showed good predictive power (R^2^ > 0.99) and non-significance of the lack-of-fit. Approximating 2.46 km h^−1^ forward speed, 50.8 mm pulley diameter, and 75 mm cutting height appeared to be optimal for providing cutting edge through cut conveyed material thus indicating the significance of coordination in kinematics between cutting and conveying processes. The developed system provides an efficient and sustainable mechanized solution to the problem of harvesting of fenugreek. These results suggest the value of crop-specific design and parameter optimization to promote precision mechanization to small-scale horticultural systems^18,20,32^.

## Data Availability

The datasets used and/or analysed during the current study available from the corresponding author (first author) on reasonable request.
